# The Impact of Peroxiredoxin 3 on Molecular Testing, Diagnosis, and Prognosis in Human Pancreatic Ductal Adenocarcinoma

**DOI:** 10.3390/cancers17132212

**Published:** 2025-07-01

**Authors:** Anna Kakehashi, Shugo Suzuki, Yusaku Nishidoi, Atsushi Hagihara, Hiroko Ikenaga, Masayuki Shiota, Guiyu Qiu, Ikue Noura, Yuko Kuwae, Arpamas Vachiraarunwong, Masaki Fujioka, Min Gi, Norifumi Kawada, Hideki Wanibuchi

**Affiliations:** 1 Department of Molecular Pathology, Osaka Metropolitan University Graduate School of Medicine, 1-4-3 Asahi-machi, Abeno-ku, Osaka 545-8585, Japan; shugo@omu.ac.jp (S.S.); m20mc008@yf.osaka-cu.ac.jp (Y.N.); k22756h@omu.ac.jp (G.Q.); sx23713o@st.omu.ac.jp (I.N.); arpamas.vachi@omu.ac.jp (A.V.); fujioka-s@omu.ac.jp (M.F.); mwei@omu.ac.jp (M.G.); wani@omu.ac.jp (H.W.); 2 Department of Hepatology, Osaka Metropolitan University Graduate School of Medicine, 1-4-3 Asahi-Machi, Abeno-ku, Osaka 545-8585, Japan; hagy@omu.ac.jp (A.H.); hiroko.ikenaga@omu.ac.jp (H.I.); kawadanori@omu.ac.jp (N.K.); 3 Department of Molecular Biology of Medicine, Osaka Metropolitan University Graduate School of Medicine, 1-4-3 Asahi-Machi, Abeno-ku, Osaka 545-8585, Japan; ms.shiota@omu.ac.jp; 4 Department of Pathology, Ishikiriseiki Hospital, Osaka 579-8026, Japan; y-kuwae@ishikiriseiki.or.jp; 5 Department of Environmental Risk Assessment, Osaka Metropolitan University Graduate School of Medicine, 1-4-3 Asahi-Machi, Abeno-ku, Osaka 545-8585, Japan

**Keywords:** PDAC, peroxiredoxin 3, oxidative stress resistance, mitochondria, extracellular vesicles, biomarker

## Abstract

**Background/Objective:** Pancreatic ductal adenocarcinoma (PDAC) is one of the leading causes of cancer death and tumors with an extremely poor prognosis. In the present study, novel biomarker candidates useful for the early diagnosis and prognosis of human invasive PDAC were investigated. **Methods:** Biomarker candidates were first selected based on the proteomic/bioinformatic and clinico-pathological analyses of 10 and 100 patients with PDAC, respectively, operated at Osaka Metropolitan University Hospital (Exp. 1). Next, the expression and secretion of the target protein and its EV mRNA were investigated in pancreatic cancer cells in vitro and in a Balb/c nude mouse model. In addition, the protein and EV mRNA levels of candidate molecules were measured in the blood serum of 36 PDAC and 10 IPMN patients, and diagnostic significance was assessed (Exp. 2). **Results:** A significant elevation of peroxiredoxin 3 (PRX3), a mitochondrial matrix protein, was found in PDAC via LC-Ms/Ms analysis. In Exp. 1, PRX3 overexpression was found in PDAC and PanIN lesions and was associated with a tumor infiltrative growth pattern (INFc) and poor overall 1-year patient survival. The prognostic value was significantly improved when PRX3 was combined with serum SPan-1 and DUPAN-2 markers in survival analyses. Furthermore, the PRX3 protein and its extracellular vesicle (EV: exosome and oncosome)-incorporated mRNA were secreted at detectable levels from PANC-1, MIAPaCa-2, and SW1990 cells into the blood of Balb/c nude mice bearing tumors. The overexpression of PRX3 was positively correlated with that of cancer stem cell marker CD44 variant 9 (CD44v9), P-Nrf2, and FOXO3a, as well as the generation of reactive oxygen species. In Exp. 2, a significant increase in PRX3 protein and EV mRNA was detected in the blood serum of PDAC subjects compared to IPMN patients and healthy controls. Significantly higher PRX3 protein levels were found in the IPMN group. The elevation of PRX3 EV mRNA was significantly associated with poor patient survival. **Conclusions:** These results indicate that PRX3 may become a novel early biomarker for PDAC diagnosis and prognosis.

## Simple Summary

In the present study, novel diagnostic and prognostic biomarkers of pancreatic ductal adenocarcinoma (PDAC) were investigated. Significant overexpression of peroxiredoxin 3 (PRX3), a mitochondrial protein involved in oxidative stress resistance, was found in PDAC lesions. Its protein and extracellular vesicle (EV)-incorporated mRNA were secreted from pancreatic cancer cells and elevated in the blood serum of PDAC patients. Furthermore, higher blood levels of PRX3, especially its EV mRNA, were associated with cancer progression, metastasis, and poor patient survival. The detection of PRX3 in PDAC may help with prognostic stratification if applied in combination with Span-1 and DUPAN-2. PRX3 protein and EV mRNA are novel early biomarkers for PDAC diagnosis and prognosis refinement.

## 1. Introduction

Pancreatic ductal adenocarcinoma (PDAC) is one of the most aggressive human malignancies, and it is difficult to detect at an early stage. In Japan, the number of deaths due to cancer is approximately 380,000 per year, of which 30.1 and 28.7 deaths per 100,000 people per year are caused by pancreatic cancer in men and women, respectively [1]. In terms of death-causing cancer, pancreatic ductal adenocarcinoma ranks fourth in men and third in women, and it is a tumor with an extremely poor prognosis, with a 5-year survival rate of about 5% [2,3]. Mutations in the KRAS gene are largely involved in the development of pancreatic cancer and activate signal pathways, such as Ras/Raf/MEK/ERK, PI3K/AKT, and RalGDS, to promote cell proliferation, enhance sugar metabolic pathways, and promote cancer formation and progression by controlling reactive oxygen species (ROS) [4,5]. The clinical symptoms include abdominal pain and early bloating, and a substantial proportion of pancreatic cancers are discovered after they have become advanced cancers. Histopathologically, approximately 90% of pancreatic cancer cases are invasive pancreatic ductal carcinoma (PDAC), which often occurs in the pancreatic head. Blood pancreatic enzymes, such as pancreatic amylase, lipase, elastase, and trypsin, are measured as blood markers, but they are not specific to pancreatic cancer. Additional tumor markers include CA19-9, CEA, DUPAN-2, and SPan-1, among others, which have a detection sensitivity for pancreatic cancer of 70–80%, 30–60%, 50–60%, and 70–80%, respectively [6,7,8,9]. Unfortunately, the positive rate of these tumor markers is low, except in advanced cancers, and their usefulness in the early diagnosis of pancreatic cancer is limited. Even in diagnostic imaging, such as CT and abdominal ultrasound, the detection rate of early pancreatic tumors of 1 cm or less is low, and the 5-year survival rate after surgery is known to exceed 80%. This tumor is difficult to diagnose and extremely difficult to detect in its early stages [2,10]. For these reasons, there is a strong desire for the development of biomarkers that contribute to the early detection and diagnosis of PDAC. In pancreatic cancer, which has a poor prognosis, prognostic markers are thought to be useful for selecting treatment strategies, including CA19-9, which is also used for diagnosis, and microRNAs miR-105, miR-127, miR-518a-2, miR-187, miR-30a-3p, SMAD4, and osteopontin [11,12,13]. However, not all are used in clinical practice, and the development of new practical diagnostic and prognostic markers is desirable [14].

Tissue-specific molecules, such as proteins and RNAs, are considered promising biomarkers found in the blood or tissue samples of patients with malignant disease. In order to be useful biomarkers for the diagnosis of small malignant lesions at a stage where curative treatment is possible, it is important for molecules to not only be highly overexpressed in tumors but also actively secreted in the blood. Proteins are expected to be released into the bloodstream due to frequent necrosis following uncontrolled tumor growth [15]. In addition to proteins, cancer cells can also secrete cell-membrane-coated extracellular vesicles (EVs): small exosomes and large oncosomes that may contain specific molecular markers [15].

**Figure 1:**
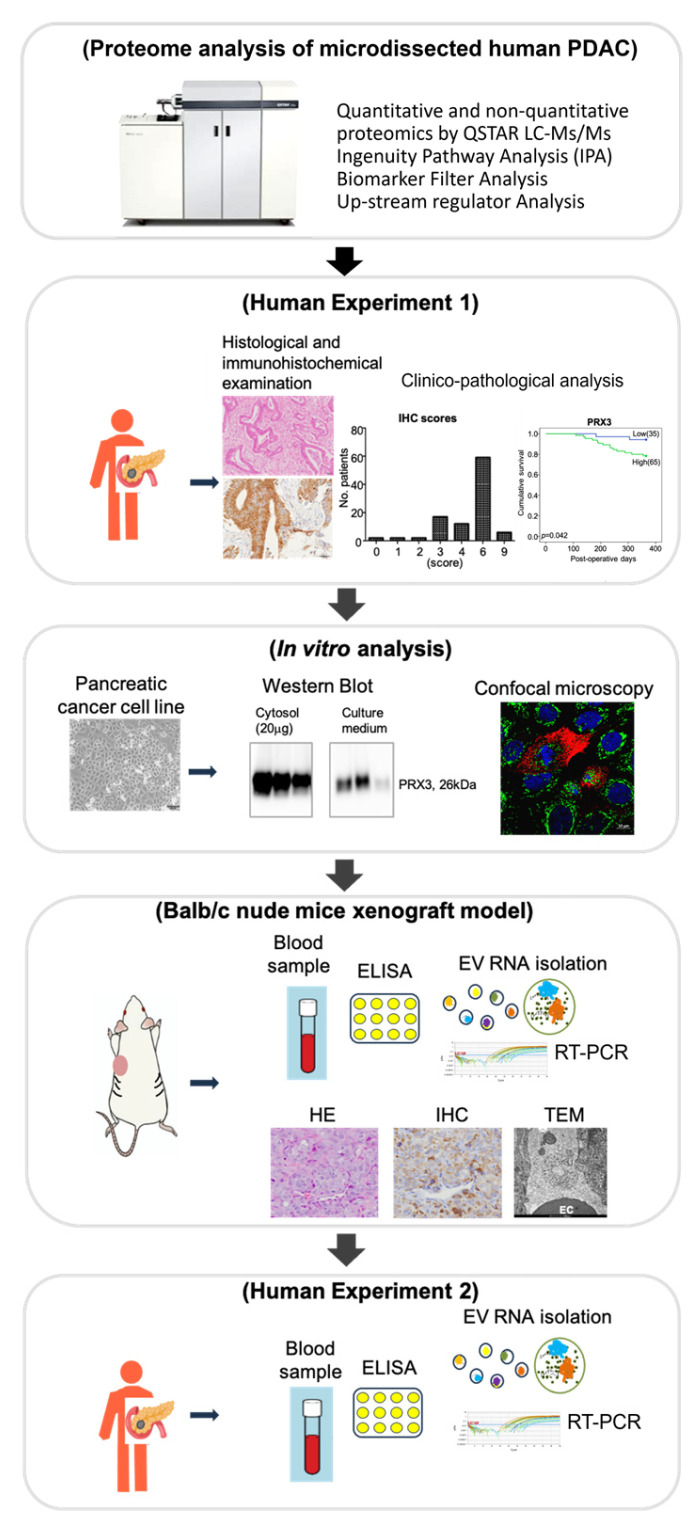
Schematic workflow. Quantitative proteomic, bioinformatic, and clinico-pathological analyses were performed on PDAC patients. Biomarker selection was performed in vitro and in vivo. Target RNA proteins and extracellular vesicles (EVs) were analyzed in the serum of patients with PDAC and IPMN and healthy control volunteers.

We have previously developed a sensitive method combining laser microdissection and LC-MS/MS for the detection of alterations in protein expression and performed a comprehensive proteomic analysis in human invasive PDAC pathological formalin-fixed paraffin-embedded (FFPE) specimens [16,17]. The aim of the present study is to identify novel biomarkers of PDAC based on the results of comprehensive proteomic, bioinformatic, clinico-pathological, in vitro, and in vivo analyses, focusing on their impact on molecular testing, diagnosis, and prognosis stratification. The procedure is schematically outlined in Figure 1. 

## 2. Materials and Methods

### 2.1. Chemicals

All reagents were obtained from Wako Pure Chemicals Industries (Osaka, Japan) and Sigma (St. Louis, MO, USA).

### 2.2. Human Tissue Specimens, Patients, Treatment, and Institutional Review Board Approval

Experiment 1. This prospective study assessed the clinical significance of expression levels of PRX3 in tissue specimens obtained from 100 patients with resected and histologically proven invasive pancreatic ductal cancer (PDAC) at Osaka Metropolitan University Hospital (Osaka, Japan) from January 2007 to December 2019. This study was approved by the Ethics Committee of Osaka Metropolitan University Graduate School of Medicine (No. 2020-164, 14 September 2024). At least two pathologists from the Department of Pathology in our hospital were enrolled in the pathological diagnoses.

Regarding clinical pathological findings, T classification, N classification, and stage classification were reclassified according to the WHO classification criteria (5th edition). Information on the features of the patients, such as age, gender, body mass index (BMI), tumor characteristics, and treatment, was obtained from medical records (Table S1). There were 47 male and 53 female patients, with a median age of 70 years (range, 40–87 years) at the time of surgery.

Experiment 2. In this study, the diagnostic significance of PRX3 protein and EV mRNA was assessed in the blood serum of 36 cases of invasive PDAC (15 males and 21 females; median age: 68.3 ± 10.1), 10 cases of intraductal papillary mucinous neoplasm (IPMN) (3 males and 7 females; median age: 72.3 ± 10.2), and 5 controls (2 males and 3 females; median age: 65.8 ± 10.2) at Osaka Metropolitan University Hospital from October 2013 to October 2024. Serum from patients with PDAC, IPMN, or controls was collected at the time of diagnosis. Control serum samples were from healthy donors. Information on the features of the patients was obtained from medical records and is presented in Table S1. Patients’ characteristics in the healthy control and experimental groups were matched regarding age and gender. No confounders were excluded. All specimens were used in compliance with the institutional review board. The study was approved by the Ethics Committee of Osaka Metropolitan University Graduate School of Medicine (No. 2024-20, 19 April 2024).

### 2.3. Selection of Candidate Proteins Through Proteome and Bioinformatic Analyses

Proteome analyses were performed with dissected PDACs and non-tumor tissue samples fixed in 10% phosphate-buffered formalin and embedded in paraffin (FFPE), as previously described [16]. Shortly, samples (20 μg each) were applied using a DiNa-AI nano LC System (KYA Technologies, Tokyo, Japan) coupled to a QSTAR Elite hybrid mass spectrometer (AB Sciex, Concord, ON, Canada) through a NanoSpray ion source (AB Sciex). After confirmation by histo-pathological analysis, needle dissection of tumors and non-tumorous areas was carried out. Samples were then dissolved in a solution optimized for the proteome analysis of FFPE sections (20 mM Tris-HCl buffer, pH 8, with 0.002% Zwittergent), heated to 90°C for 90 min, and digested with trypsin at 37 °C for 16~18 h. Labeling was performed using 4-plex iTRAQ reagents in quantitative analysis. The samples were labeled with iTRAQ isobaric reagents for both PDAC and non-tumor pancreas tissues and then refined by cation exchange column chromatography. The peptides were eluted as 6 fractions (1 mL of KCl solution of 10, 50, 70, 100, 200, and 350 mM), and the supernatants were evaporated using a vacuum centrifuge. After desalting and concentration processes with Sep-Pak columns (WATO 23501 Light C18), the samples were resuspended in 20 μL of 0.1% (vol/vol) formic acid and applied onto LC-MS/MS. The separation of samples was performed isocratically, 95A/5B *v*/*v* (A:98%, water/2%, ACN/0.1% formic acid; B: 30%, water/70%, ACN/0.1% formic acid). Each sample was run for 150 min. The data were searched against the Swiss Protein Database (HUMAN) using ProteinPilot Software 2.0 (AB Sciex, Tokyo, Japan) with trypsin set as the digestion enzyme and methyl methanethiosulfonate as the cysteine modification. The reported data were used at a 95% confidence cut-off limit. Protein levels were quantified by comparing iTRAQ reporter ion intensities between PDAC and non-tumor samples with a *p* < 0.05. To assess the validity of the protein expression changes, after the 1-sample *t* test of the averaged protein ratio against 1, a *p*-value was reported.

To find the potential biomarker candidates and altered pathways in PDAC, the bioinformatic analysis of the LC-MS/MS data of 10 invasive PDAC cases (AB Sciex, Concord, ON, Canada) [16] was performed using Ingenuity Pathway Analysis (IPA) 24.0 software (Ingenuity Systems, Mountain View, CA, USA). Candidate biomarker proteins were selected based on their expression level (more than 5-fold), biomarker filter analysis using Ingenuity Pathways Analysis, and detailed information on protein molecular functions, intracellular localization, and the possibility of excretion from cancer cells. The analyses of upstream regulators and canonical pathways aimed to identify the common transcriptional factors that may be driving the protein expression changes; their activity was measured using the z-score; values above 2 were considered significant. The proteins included in the biomarker filter list were subjected to immunohistochemical and clinico-pathological analyses, and analysis of mRNA expression in extracellular vesicles was conducted in a Balb/c nude mouse model and in the blood of the PDAC patients.

### 2.4. Immunohistochemical Examination

#### 2.4.1. Peroxiredoxin 3 (PRX3) Immunohistochemistry and Scoring

Immunohistochemical staining was performed on PDAC FFPE sections using the ABC method with the VECTASTAIN Flite ABC kit (Vector Laboratories, Burlingame, CA, USA), using antigen retrieval via citrate buffer (10 mM, pH 6.0) for 20 min at 98 °C and inactivation of endogenous peroxidase by 3% hydrogen peroxide. Reaction with rabbit monoclonal antibody for PRX3 (1:250, ab128953, Abcam, Tokyo, Japan) (human: P30048; mouse: P20108) was performed overnight at 4 °C. Positive regions were stained with 3,3-diaminobenzidine tetrahydrochloride (DAB; Dojindo Laboratories, Inc., Kumamoto, Japan). Immunohistochemical specimens were evaluated for the staining intensity of PA cells (1: weakly positive; 2: positive; 3: strongly positive). The scores were evaluated as the proportion of tumor cells with different levels of staining intensity in the tumor tissue (1: ≤ 10%; 2: 11–50%; 3: >50%). For each specimen, the IHC scores were calculated based on the staining intensity and the proportion of positive tumor cells in the specimen (from 1 to 9). For each sample, IHC scores from 1 to 4 were weakly positive (Low), and those from 5 to 9 were strongly positive (High) using the median split dichotomization method, which was considered most appropriate to describe the present IHC results.

#### 2.4.2. Immunohistochemical Assessment of CD44v9, P-Nrf2, p62, P-Foxo3a (Ser7, Ser574, Ser253), and Ki67, and Double Staining for CD44v9, Ki67, and PRX3

For specimen processing, single and double immunohistochemistry for CD44v9, phosphor-Nrf2 S40 (P-Nrf2), p62, FOXO3a, phosphor-FOXO3a (S7, S253, and S574), Ki67, and PRX3 were performed using the ABC method, as described previously [17]. In brief, following deparaffinization and rehydration, microwave antigen retrieval was performed in citrate buffer (10 mM, pH 6.0) for 20 minutes at 98 °C. Endogenous peroxidase activity was blocked by immersing the slides in 3% (*v*/*v*) hydrogen peroxide for 5 min. Each section was incubated overnight at 4 °C with the primary rabbit monoclonal antibody against P-Nrf2 Ser40 (1:250, SAB5701902, Sigma Aldrich, St. Louis, MO, USA), rabbit polyclonal antibody to p62 (1:300, PM-045, MBL, Tokyo, Japan), rabbit monoclonal antibody against FOXO3a D19A7 (1:1000, #12829, Cell Signaling, Danvers, MA, USA) and P-FOXO3a Ser253 (1:150, 9466, Cell Signaling Technology, Danvers, MA, USA), rabbit polyclonal antibody to P-Foxo3a-Ser7 (1:130, #PA5-104595, Invitrogen, Carlsbad, CA, USA) and P-FOXO3a-Ser574 (1:100, #86-8052-88, Signaling Antibody, Tokyo, Japan), and rat monoclonal anti-human antibody against CD44v9 (1:300, RV3 LKG-M001, Cosmo Bio Co., Ltd., Tokyo, Japan). Ki67 was detected with a rabbit monoclonal antibody (MIB-1) (1:25, SC-101861, Santa Cruz Biotechnology, Inc., Dallas, TX, USA) at 4 °C. For singles, IHC, PRX3, P-Nrf2, FOXO3a, P-FOXO3a (Ser253, Ser7, and Ser574), CD44v9, and Ki67 were detected using a Vectastain Elite Kit (Vector Laboratories, Burlingame, CA, USA) and a 3,3′-diaminobenzidine tetrahydrochloride (DAB) solution (DAKO, Tokyo, Japan). The sections were counterstained with hematoxylin (Sigma-Aldrich, Inc., St. Louis, MO, USA), dehydrated, and mounted. All immunohistochemical slides were analyzed by two pathologists independently. Both expression levels and the localization of target molecules were analyzed and interpreted using guidelines published in previous studies [18]. Nuclear P-Nrf2 was graded from 0 to 2+ as follows: 0, no staining; (1, ≤10%; 2, 11–50%, 3, >50%). For membranous CD44v9 and FOXO3a, the results were graded from 0 to 3+ as follows: 0, no staining; 1+, 1–25% staining; 2+, 26–50% staining; 3+, >50% of the specimen was stained.

In addition, double staining for CD44v9 and PRX3 and Ki67 and PRX3, using the same antibodies and the dilutions as in the single immunohistochemistry, was performed using DAB and alkaline phosphatase (Vectastain ABC-AP kit, Vector Red (SK-5100), Tokyo, Japan) for the detection of CD44v9, Ki67, and PRX3, respectively. CD44v9 and Ki67 were stained brown/black, while PRX3 was stained red. A negative control was included in every staining and was processed with primary serum instead of antibodies.

### 2.5. In Vitro Experiments

#### 2.5.1. Cell Lines and Culture Conditions

The pancreatic adenocarcinoma cell lines (PANC-1, MIAPaCa-2, SW1990, and RWP-1) were obtained from the American Type Culture Collection (ATCC) (Manassas, VA, USA). PANC-1, SW1990, and RWP-1 were maintained in RPMI-1640 medium (FujiFilm Co., Osaka, Japan), supplemented with 1% penicillin/streptomycin (Wako Pure Chemical Industries, Ltd., Osaka, Japan) and 10% fetal bovine serum (FBS; Hyclone Laboratories Inc., Logan, UT, USA). MIAPaCa2 cells were cultured in Dulbecco’s modified Eagle’s medium (DMEM) (Wako, Osaka, Japan) with 1% penicillin/streptomycin (P/S) and 10% FBS. All cells were incubated at 37 °C in a 5% CO_2_ air-humidified atmosphere.

#### 2.5.2. PRX3 Knockdown in PANC-1, MIAPaCa-2, and SW1990 Cells

Knockdown of PRX3 was carried out with 2 PRX3 siRNAs and confirmed by RT-PCR and Western blot analyses. Western blot demonstrated a significant reduction in protein level of 26 kDa PRX3 protein compared with the siRNA control samples in the PANC-1, MIAPaCa-2, and SW1990 cell lines transfected with PRX3 Silencer Pre-designed siRNAs 1 (Cat. #: AM16708, ID: 136071, Invitrogen, Tokyo, Japan) and 2 (ID:18510) with Lipofectamine RNAiMAX (Invitrogen, Tokyo, Japan), according to the manufacturer’s instructions. The best PRX3 knockdown was obtained with PRX3 siRNA-2. All reactions were performed in triplicate.

#### 2.5.3. Estimation of Reactive Oxygen Species (ROS) Levels After PRX3 Knockdown

In order to explore the effect of PRX3 knockdown on oxidative stress in pancreatic cancer cells, we carried out an estimation of ROS levels using the Highly Sensitive DCFH-DA-ROS assay kit (#18606, Dojindo Laboratories, Kumamoto, Japan) according to the manufacturer’s instructions. Briefly, approximately 1 × 10^4^ cells/100 μL assay solution was transferred to assay wells in a 96-well assay plate and incubated at 37 °C and 5% CO_2_ for 24 h, with each group being assayed in triplicate. Cells were incubated for 72 h after the addition of PRX3 siRNA or the negative control. At termination, cells were washed twice with Hank’s Balanced Salt Solution (HBSS) and incubated with 100 μL highly sensitive H2DCFH-DA dye working solution for 30 min. Following washing twice with HBSS, the fluorescence intensity measurements were carried out at an excitation wavelength of 490 nm and an emission wavelength of 520 nm using the fluorescence microplate reader (Varioskan LUX, Thermo Fisher Scientific Inc., Waltham, MA, USA). The numbers were presented as fluorescence intensity ratio vs. untreated cells per well and plotted as mean ± SD. All reactions were performed in triplicate.

#### 2.5.4. Analysis Using the Confocal Microscopy

Double immunohistochemistry for PRX3 and CD44v9 and confocal microscopy were performed in PANC-1, SW1990, RWP-1, and MIAPaCa-2 cells. A total of 5 × 10^4^ cells/well were seeded in a 24-well plate and incubated in RPMI-1640 or DMEM medium, with 10% FBS and 1% P/S. Cells were washed with PBS and fixed in 3% paraformaldehyde (PFA)/PBS for 20 min. Then, cells were washed with PBS for 10 min, and the permeabilization was performed with 0.1% Triton X-100/PBS for 5 min. Afterward, cells were washed and incubated with 1% BSA and 10% goat serum/PBS and exposed to primary rat monoclonal anti-human antibodies against CD44v9 (1:300, RV3 LKG-M001, Cosmo Bio Co., Ltd., Tokyo, Japan) and PRX3 (1:500; ab128953, Abcam, Tokyo, Japan) in 50% blocking serum/PBS for 1 h. Next, cells were washed and incubated with secondary antibodies, rabbit-IgG Alexa488 for PRX3 (1:1000, A-11008, Invitrogen, Carlsbad, CA, USA), rat-IgG Alexa Fluor 568 (1:1000, ab175476, Abcam, Tokyo, Japan) for CD44v9, and DAPI (1:2000, Biotium Inc., Fremont, CA, USA) in 50% blocking serum/PBS for 40 min, and then washed and desalted in distilled water. The coverslips were mounted using FluorSave Reagent (EMD Millipore, Tokyo, Japan) and microcoverglasses (12 mm CIR-180, Matsunami Corp., Osaka, Japan). Fluorescence imaging was performed using a Zeiss LSM-900 confocal laser scanning microscope (Zeiss, Tokyo, Japan). Images were acquired using ZEN 2.1 Blue Edition Gentle Multiplex Imaging software (Zeiss). Examinations were performed in triplicate.

#### 2.5.5. Protein Extraction from Culture Supernatant and Pancreatic Cancer Cells

PANC-1, RMP-1, and SW1990 pancreatic cancer cell lines were cultured for 2 days in RPMI-1640 medium with 10% FBS and 1% penicillin-streptomycin. Thereafter, the culture supernatant was replaced with FBS and P/S-free medium, and cells were cultured for another 2 days. The collected culture supernatant was ultrafiltered using an Amicon 3k tube (Amicon^®^ Ultra: Merck KGaA, Darmstadt, Germany) and used for Western blot analysis. Cells were collected, and the lysis was carried out with Radio-Immunoprecipitation Assay (cRIPA) buffer with 50 mM Tris-HCl (pH 7.4), 150 mM NaCl, 1 mM EDTA (pH 8.0), 1% NP-40, 0.5% sodium deoxycholate, 0.1% SDS, 100 mM phenylmethylsulfonyl fluoride, and protease inhibitor cocktail set (FujiFilm, Wako, Osaka, Japan). The collected cell suspension was subjected to ultrasonication and centrifugation at 15,000 rpm for 15 min. The protein concentration of the culture supernatant and cell protein extract was measured using a BCA Protein Assay Kit (FujiFilm Wako Pure Chemical Industries, Ltd., Osaka, Japan). Examinations were performed in triplicate.

#### 2.5.6. Western Blot

Sample protein extracts were prepared in 6x sample buffer, followed by denaturing treatment at 65 °C for 5 min. Electrophoresis was carried out on 12.5% polyacrylamide gels, which were blotted on PVDF membranes (Immobilon-P membrane; Merck KGaA, Darmstadt, Germany). After the transfer, the membrane was blocked for 60 min at room temperature using 5% skim milk (Morinaga Milk Products Co., Ltd., Tokyo, Japan) and exposed to the PRX3 rabbit monoclonal primary antibody diluted with 1% skim milk solution (1:2500, ab128953, Abcam, Tokyo, Japan) at 4 °C overnight. After washing with Tris-base saline buffer with 0.1% Tween 20 (TBS-T), a secondary goat anti-rabbit IgG-HRP antibody (1:5000, sc-2054, Santa Cruz Biotechnology, Inc., Dallas, TX, USA) was applied for 60 min at room temperature. Then, membranes were stained with HRP coloring solution (SuperSignal™ West Pico PLUS Chemiluminescent Substrate; Thermo Fisher Scientific, Waltham, MA, USA) for 1 min at room temperature, and the reaction was detected using Fusion SOLO.7S (Vilber Lourmat, Collégien, France). All reactions were carried out in triplicate.

### 2.6. Quantitative Reverse Transcription Polymerase Chain Reaction (RT-PCR)

Total RNA was isolated from prefiltered patient or mouse blood serum/plasma using the exoRNeasy Midi Kit (Cat# 77144, Qiagen, Hilden, Germany) according to the manufacturer’s instructions. RNA was isolated from prefiltered blood samples to obtain EVs with sizes of <800 nm. The exoRNeasy protocol provides higher purity isolations and is faster than ultracentrifugation. Scanning electron microscopy has shown that both exoEasy and ultracentrifugation yield intact EVs of the expected size, but ultracentrifugation also yields smaller structures that differ in size and shape. cDNA synthesis was carried out using a ReverTraAce qPCR RT Master Mix (Code: FSQ-201, Toyobo, Osaka, Japan). The mRNA expression levels of PRX3, Nrf2, p62 FOXO3a, and CD44v9 were determined by TaqMan real-time quantitative PCR using TaqMan probes (PRX3: Hs00428953_g1, NFE2L2 (Nrf2): Hs00975961_g1, SQSTM1 (p62): Hs00177654_m1; FOXO3: Hs00818121_m1 (Cat # 4331182), and CD44: Hs01075857_m1 (Cat #,4351372) (Life Technologies, Tokyo, Japan). PCR reagents and sequence-specific primers and probes for each gene (Taqman Gene Expression Assay) were purchased from Thermo Fisher Scientific Inc. mRNA expression assays were carried out using a 7500 Fast Real-Time PCR System (Thermo Fisher Scientific Inc., Tokyo, Japan). 18S ribosomal RNA was an internal control. Values for target genes were normalized to those for 18S. All reactions were performed in triplicate.

### 2.7. Detection of PRX3 Plasma/Serum Levels by ELISA

PRX3 blood plasma and serum levels were measured using a commercially available kit (Human PRDX3 ELISA (Cat# OKEHO02204, Aviva System Biology, San Diego, SA, USA) according to the manufacturer’s instructions. In brief, 100 μL of each dilution of standard (20, 10, 5, 2.5, 1.25, 0.625, and 0.313 ng/mL) was taken and diluted 30 times; the samples were added to the ELISA plates and incubated for 2 h at 37 °C. Thereafter, 100 μL of biotinylated PRX3 detector antibody was added to wells, and incubation was performed for 1 h at 37 °C. After washing, samples were incubated with Avidin-HRP conjugate for 1 h at 37 °C. TMB substrate solution was added after washing, followed by incubation for 20 min at 37 °C. The reaction was terminated by the addition of a stop solution. Absorbance was measured spectrophotometrically at 540 nm using a model 680 microplate reader (BIO-RAD, Tokyo, Japan). A standard curve was drawn for each plate, and PRX3 concentrations were determined by comparing their optical density (OD) with the standard curve after the subtraction of the OD value for the background. All reactions were performed in triplicate.

### 2.8. In Vivo Nude Mice Xenograft Model

The animal study was performed in accordance with the Guidelines of the National Institute of Health and Public Health Service Policy on the Humane Use and Care of Laboratory Animals and approved by the Ethics Committee of the Institutional Animal Care and Use Committee of Osaka Metropolitan University Graduate School of Medicine, Osaka, Japan (No. 23056). Five-week-old female Balb/C nu/nu mice (CAnN.Cg-Foxn1nu/CrlCrlj) were purchased from Charles River Co. (Hino, Shiga, Japan). Mice were divided into 4 experimental groups (10 mice per group). Pancreatic cancer cells (PCCs) (PANC-1 (1 × 10^6^), MIAPaCa-2 (3 × 10^6^), and SW1990 (1 × 10^7^)) were suspended in 200 μL of Matrigel and inoculated subcutaneously into the back of 6-week-old nude mice. In all cases, the injected cells developed a subcutaneous tumor. The diameter of tumors was measured every 3–4 days. When tumors reached 20 mm in diameter, the mice were euthanized under isofluorane. The blood was collected from the abdominal vein, and plasma was obtained, immediately frozen, and kept at −80 °C. Tumors were examined macroscopically, weighed, and partly fixed in 10% formalin and 2% glutaraldehyde/2% paraformaldehyde solutions for histopathological, immunohistochemical (IHC), and transmission electron microscopy (TEM) analyses performed using the standard protocols.

### 2.9. Statistical Analysis

Statistical analyses were performed using SPSS Statistics version 19.0 (SPSS Inc., Chicago, IL, USA) and GraphPad Prism 8 software (GraphPad Software, San Diego, CA), and all *p*-values were 2-sided. The area under the receiver operating characteristic curve (ROC AUC) and Yunden indices were calculated for testing the discrimination ability of the biomarker, and the 95% confidence interval (95% CI) was estimated. A χ^2^ test (Fisher exact test) was carried out to find the association with clinical variables (age, sex, smoking, alcohol consumption, DM, tumor markers, pathological findings, and Ki67, P-Nrf2, CD44v9, and FOXO3a status). PRX3, weakly or strongly positive in PDAC cases, and for PRX3 in combination with serum pancreatic cancer tumor markers SPan-1 and DUPAN-2, were created using the Kaplan–Meier method from the date of surgery to the date of death or the last follow-up observation using SPSS statistics and evaluated by the log-rank test. Univariate and multivariate Cox proportional hazard model analyses were carried out for the calculation of hazard ratios (HRs) and the determination of associations between clinico-pathologic variables and disease-specific mortality. In all tests, *p* < 0.05 was considered statistically significant.

## 3. Results

### 3.1. Clinical Findings of Patients Targeted for Immunohistochemistry and Blood Analyses

The clinical and pathological findings of PDAC and intraductal papillary mucinous neoplasm (IPMN) patients subjected to histopathological, IHC, and blood analyses are presented in Table S1. The gender and age of all patients were adjusted within the PDAC, IPMN, and control groups. The exclusion criteria included the following: cases with active double cancers with a disease-free interval of 5 years or less; cases with serious complications that are considered to be more prognostic factors than invasive PDAC; women who may be pregnant or breastfeeding; patients who have undergone preoperative CRT; and cases with a follow-up period of less than 1 year.

In Experiment 1, 100 PDAC patient tumor tissues were subjected to the histopathological and IHC analyses of PRX3, phospho-Nrf2 (P-Nrf2 (S40)), CD44 variant 9 (CD44v9), and FOXO3a. The mean age was 70.0 ± 8.1 (40–87) years, and there were 47 male and 53 female patients. The post-operative follow-up period ranged from 109 to 2881 days (median: 689 days), and 60 patients died. During the observation period, 30 patients experienced recurrence, and 47 patients had metastasis. Among the prepared specimens, lesions equivalent to PanIN (hyperplastic lesions at least 1.0 cm from the tumor site) were observed around the tumor lesion in 4 out of 100 cases. Of the 100 PDAC patients subjected to IHC analysis, 60 (60%) had tumors localized in the pancreas head, and 8 (8%) were in the pancreas tail. The tumor size was more than 2 cm in diameter in 77 (77%) patients, with frequent lymphatic and venous invasion in 65 (65%) and 38 (38%) cases, respectively. Diabetes was observed in 34 (34%) PDAC patients.

In Experiment 2, 36 PDAC, 10 IPMN, and 5 control subjects were subjected to blood serum analyses for the evaluation of the PRX3 protein via ELISA, and PRX3, Nrf2, CD44v9, FOXO3a, and p62 mRNA from EVs were evaluated using RT-PCR. The mean age of the patients whose blood samples were analyzed was 64.4 (55–85), 72.3 ± 10.2 (55–87), and 65.8 ± 10.2 (52–78) years in the PDAC, IPMN, and control groups, respectively. The PDAC, IPMN, and control groups included 15 males and 21 females, 3 males and 7 females, and 2 males and 3 females, respectively. In total, 19, 12, and 5 cases were diagnosed with PDAC stage 4 (19 (52.8%)), stage 3 (12 (33.3%)), and stage 2 (25 (80.6%)) in accordance with WHO classification, respectively. CA19-9, CEA, DUPAN-2, and SPan-1 were elevated in 76 (76.0%), 23 (25.3%), 33 (43.4%), and 59 (62.1%) PDAC patients, respectively (Table S1).

### 3.2. Bioinformatic Analysis and Selection of Candidate Biomarker Proteins

Bioinformatic analysis was performed based on proteomic data obtained via the LC-MS/MS analysis of 10 PDAC patient tumors and non-tumor, formalin-fixed, and paraffin-embedded (FFPE) samples using Ingenuity Pathway Analysis (IPA), and the results are presented in Table 1 and Tables S2–S4 [16].

Firstly, among the proteins overexpressed in cancerous areas, 103 proteins with cytoplasmic localization and more than a 5-fold elevation in the PDAC area were selected (Table S2). Then, the Biomarker Filter analysis in IPA was performed based on the examination of all potential molecular candidates with high and frequent elevation. Special attention was paid to markers potentially secreted from the tumor tissue (Table S3). One of the selected proteins via Biomarker Filter IPA was the mitochondrial matrix protein peroxiredoxin 3 (PRX3; Uniprot ID: P30048), which was strongly (8-fold) and frequently elevated in PDAC and had the potential to be secreted from tumor tissue. Proteins identified in the Biomarker Filter analysis were subjected to immunohistochemical and clinico-pathological examination and blood analysis in PDAC patients to identify the best biomarker candidate. To determine whether PRX3 and other potential candidates could be candidate biomarkers for PDAC, we performed IHC assessments and clinico-pathological analyses in 100 PDAC patients, and we investigated potential protein and mRNA secretion in vitro and in vivo using a Balb/c nude mouse xenograft model and the blood of PDAC and IPMN patients.

Upstream regulator and signaling pathway analyses revealed that significantly activated transcriptional regulators of PDAC play a central role and participate in the control of cell proliferation, the activation of fibrogenesis, angiogenesis, protection from deteriorative effects of ROS, and oxidative stress resistance. These included transforming growth factor β (TGF-β), nuclear factor erythroid 2-related factor 2 (Nrf2), nuclear factor kappa B (NF-κB), β-catenin (cadherin-associated protein, beta 1 (CTNNB1)), hypoxia-inducible factor 1 subunit alpha (HIF1A), cancer stem cell marker CD44, forkhead fox A2 (FOXA2) and A3 (FOXO3), and transcriptional factor SP1, as well as mitogen-activated protein (MAP) kinases p38, AKT, PI3K, and ERK1/2 (Table 1).

Canonical pathway analysis via IPA revealed that proteins significantly up- or downregulated in PDAC were mainly involved in fibrogenesis, actin cytoskeleton and RHOA signaling, collagen receptor glycoprotein 6 (GP6) signaling, phagosome formation, the production of nitric oxide and reactive oxygen species (ROS), and the Nrf2-associated oxidative stress response (Table S4).

### 3.3. PRX3 Staining in Human Invasive PDAC, Clinico-Pathological Analysis, and Positive Association with P-Nrf2, CD44 Splicing Variant 9 (CD44v9), and FOXO3a (Exp. 1)

**Figure 2:**
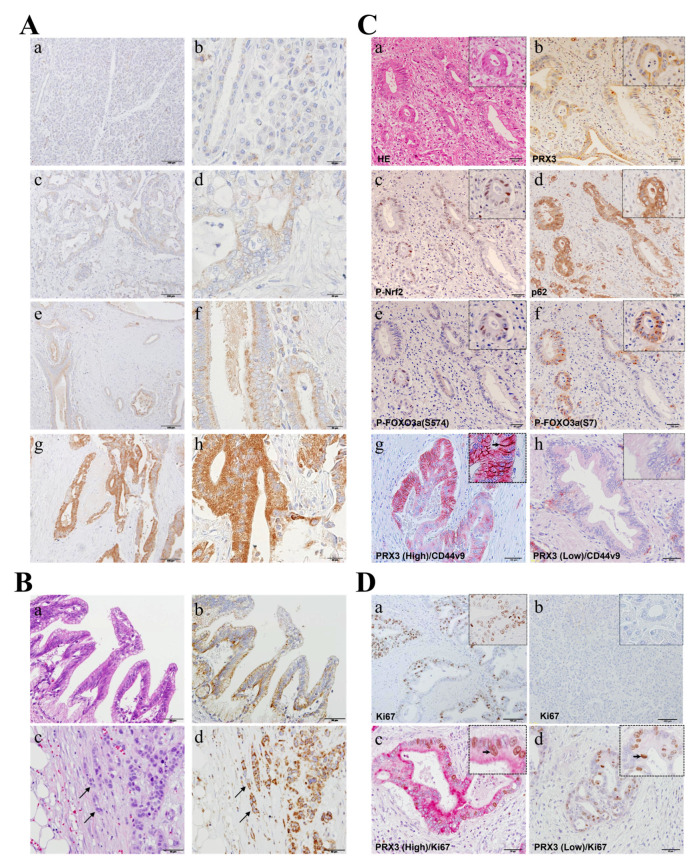
Immunohistochemical assessment of PRX3, P-Nrf2(S40), p62, P-FOXO3a(S574), P-FOXO3a(S7), and Ki67 (**Exp. 1**). (**A**) PRX3 immunohistochemistry in PDAC. PRX3 protein expression was negative in non-tumor tissue (**a,b**) and low (**c**,**d**), medium (**e**,**f**), and high (**g**,**h**) in PDAC (×100 and ×200, respectively). (**B**) PRX3-positive PanIN (**a**,**b**) and invasive PDAC (**c**,**d**) (×200). (**C**) Immunohistochemical assessment of PRX3, P-Nrf2, p62, FOXO3a(S574), and P-FOXO3a(S7) in serial sections of PDAC (**a**–**f**) and double staining for PRX3 and CD44v9 (PRX3: red (cytosol); CD44v9: brown/black (cell membrane)) (**g**,**h**) (×200); (**D**) Ki67 staining in tumor (**a**) and non-tumor (**b**) areas (×100) and double immunohistochemistry for Ki67 and PRX3 in regions with high (**c**) and low (**d**) expression (PRX3: red (cytosol); Ki67: brown/black (nuclei)) (×200).

PRX3 expression was immunohistochemically assessed in 100 PDAC cases to evaluate its correlation with clinico-pathological variables (Figure 2). In high-power fields, granular PRX3 staining in the cytoplasm of pancreatic ductal carcinoma cells was identified within tumors (Figure 2A). IHC staining revealed PRX3-positive tumors in 98 out of 100 cases (IHC score 0: 2 cases (2%); 1: 2 cases (2%); 2: 2 cases (2%); 3: 17 cases (17%); 4: 12 cases (12%); 6: 59 cases (59%); and 9: 6 cases (6%)). In total, 35 invasive PDAC cases (35%) were weakly positive (scores of 0 to 4), and 65 cases (65%) were strongly positive (scores of 5 to 9) for PRX3. Importantly, all observed preneoplastic PanIN lesions found in non-tumor areas were positive for PRX3 (Figure 2B). Furthermore, in specimens with the INFc infiltrative growth pattern, a strong increase in PRX3 was obvious at the tip of the invasion (Figure 2B).

Next, we examined the expression of oxidative-stress-resistance-related proteins, including PRX3, phospho (P)-Nrf2 (S40), p62, P-FOXO3a (S574; activated by JNK), P-FOXO3a (S7; activated by p38/ERK), and P-FOXO3a (S253; activated by AKT) via IHC in serial sections or using double IHC and compared their expression in PDAC cancer tissues to assess the role of PRX3 in the regulation of intracellular oxidative stress (Figure 2C). It was observed that P-Nrf2, p62, and activated forms of FOXO3a transcriptional factor P-FOXO3A (S594) and P-FOXO3a (S7) were barely detectable in cells in PDAC regions with low PRX3 expression but were elevated in those with high PRX3 (Figure 2C). Furthermore, cancer stem cell (CSC) marker CD44 variant 9 (CD44v9), which is involved in oxidative stress resistance and the maintenance of glutathione (GSH) levels in tumor cells [19], was significantly overexpressed in highly PRX3-positive PA cells (Figure 2C). The CD44v9-positive cell index (the percentage of CD44v9-positive cells (brown/black)) in PRX3-high areas (red) was significantly increased (22.2 ± 21.5%) compared to that of PRX3-low areas (2.6 ± 4.6%) (*p* < 0.001).

### 3.4. Evaluation of Cell Proliferation and Its Association with PRX3 Expression

In the specimens of 100 patients examined, cell proliferation marker Ki67 IHC indices were calculated as the ratio of Ki67-positive cells (brown/black) to the total examined PA cells in PRX3-high- and PRX3-low-positive areas (red) (double IHC). As a result of examining the number of Ki67-positive cells in tumor cells, the median Ki67 index was 21.26% (3.78–67.41%) (Figure 2D). With values above the median defined as Ki67-high and those below the median defined as Ki67-low, there was no statistically significant difference between weakly and strongly PRX3-positive PDAC areas. Moreover, when we compared PRX3 and Ki67 via double IHC, both PRX3 high- and low-power fields were positive for Ki67, and no correlation was obvious between PRX3 and Ki67 expression (Figure 2D). Furthermore, clinico-pathological analyses demonstrated that there is no clear correlation between Ki67 and PRX3 expression (Table 2). From these results, PRX3 expression was considered not to be correlated with proliferation ability.

### 3.5. Association of PRX3 with Clinico-Pathological Variables (Exp. 1)

The relationship between PRX3 elevation in PDAC and various clinico-pathological variables—including tumor size, TNM factors, pathological stage, tissue differentiation, venous invasion, and the invasive growth mode of human PDAC—was analyzed using a chi-square test (Table 2).

A positive significant correlation between high PRX3 expression in PDAC and an infiltrative growth pattern (INFc vs. INFab, *p* = 0.013), CD44v9 status (positive vs. negative, *p* = 0.044), p-Nrf2 (S40) status (high vs. low, *p* = 0.003), and FOXO3a status (high vs. low, *p* = 0.0001) was found.

In additional correlation analysis, the elevation of P-Nrf2 and FOXO3a was associated with venous invasion (*p* = 0.029 and *p* = 0.035, respectively), while CD44v9 was positively correlated with recurrence and metastasis (*p* = 0.015), the INFc infiltrative growth pattern of PDAC (INFc vs. INFa,b; *p* = 0.041), T factor (pT4 vs. pT1–3; *p* = 0.036), patients’ smoking status (smoker vs. non-smoker; *p* = 0.01), and blood amylase levels (>126 U/L vs. 40–126 U/L; *p* = 0.007) (chi-square test).

### 3.6. Prognosis (Exp. 1)

**Figure 3:**
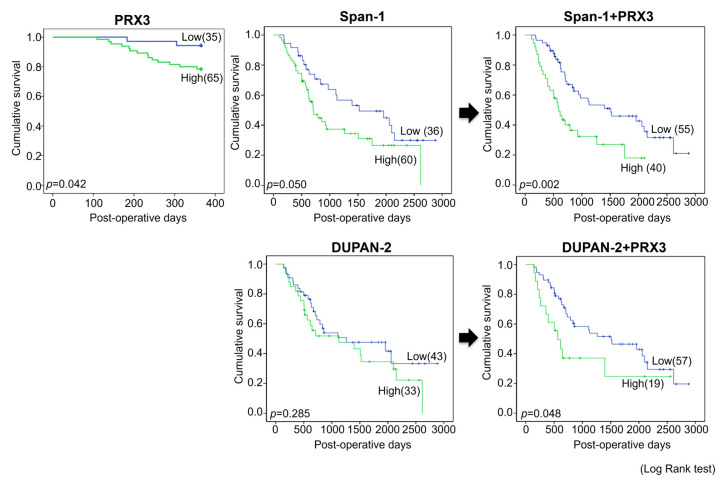
Survival curves of PRX3-, Span-1-, and DUPAN-2-positive patients (Exp. 1). The combination of PRX3 with Span-1 and DUPAN-2 significantly improved the prognosis of PDAC. The combined biomarker Low-expression group included all subjects that were low in at least one of the individual markers, which led to the variation observed in the number of subjects. The differences in overall survival were assessed using a log-rank test.

Next, univariate survival analysis with overall survival curves was performed according to the Kaplan–Meier method, and differences in overall survival were assessed with the log-rank test (Figure 3).

Significant differences in survival between PRX3-high- and PRX3-low-positive patients were found in the 1-year follow-up period, where strongly positive PRX3 cases demonstrated a worse prognosis (*p* = 0.042). However, no significant differences in the survival of PRX3-high- and PRX3-low-positive patients were detected for the entire 5-year observation period. Importantly, when PRX3 positivity was combined with blood serum Span-1 or DUPAN-2 positivity, PRX3 significantly improved the prognostic sensitivity of these two existing PDAC markers over the entire 5-year follow-up period (*p* = 0.002 and *p* = 0.048, respectively) (Figure 3). The univariate and multivariate analyses of survival correlations with clinico-pathological variables and PRX3 tissue levels are presented in Table S5. According to the results of univariate analysis, poor patient survival was significantly associated with an increase in combined PRX3 and blood serum Span-1 expression (hazard ratio (HR), 2.287, *p* = 0.003); combined PRX3 and DUPAN-2 expression (HR, 1.950, *p* = 0.049); tumor size (>4 cm vs. ≤4 cm; HR, 1.907, *p* = 0.047); lymphatic invasion (positive vs. negative; HR, 2.836, *p* = 0.011); infiltrative growth pattern (INFc vs. INFab; HR, 2.364, *p* = 0.018); arterial invasion (pA) (positive vs. negative; HR, 2.432, *p* = 0.028); tumor differentiation (poorly vs. well and moderately differentiated; HR, 2.432, *p* = 0.029); higher T category (T3,4 vs. T1,2; HR, 2.035, *p* = 0.014); pN status (N1,2 vs. N0; HR,1.648, *p* = 0.05); and clinico-pathological stage (stages 2–4 vs. 1; HR, 1.904, *p* = 0.02). It was observed that the evaluation of PRX3 expression significantly improved the prognosis of patient survival if combined with Span-1 and DUPAN-2 markers.

Multivariate analysis demonstrated that the poor survival of patients was significantly associated with lymphatic invasion (positive vs. negative; HR 0.291, *p* = 0.046); elevated CA19-9 level (>35 vs. ≤35; HR 0.207, *p* = 0.023); and combined PRX3 and blood serum Span-1 expression (high vs. low; HR 0.176, *p* = 0.013) (Table 3).

### 3.7. In Vitro Analyses

#### 3.7.1. Secretion of PRX3 Protein by PA Cells into the Culture Medium

**Figure 4:**
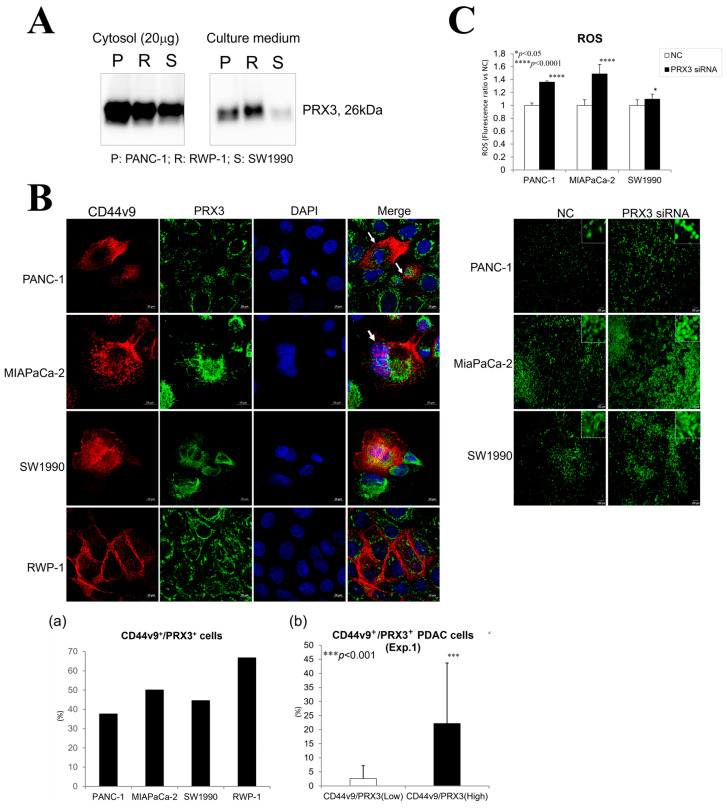
In vitro analysis of PRX3 protein secretion, association with CD44v9, and antioxidant function. (**A**) Western blot analysis of PRX3 protein levels in pancreatic cancer cell lines, cytosol, and the culture medium. Note the elevation of PRX3 protein levels in both the cytosol and the culture medium. (**B**) Double IHC for PRX3 and CD44v9 (confocal microscopy) in PANC-1, MIAPaCa-2, SW1990, and RWP-1 cells (×400). Note that there are more PRX3-single-positive cells (green (mitochondria)) and fewer double-positive cells for PRX3 (green) and CD44v9 (red (cell membrane/cytosol)). CD44v9/PRX3 double-positive cells with CD44v9 elevated in the membrane and cytoplasm were often undergoing mitosis (arrows). The highest number of double-positive cells was observed in RWP-1 cells (**a**). In Exp. 1, CD44v9-positive staining was found predominantly in the highly PRX3-positive areas of PDAC tumors. *** *p* < 0.001 vs. CD44v9/PRX3 (low) (**b**). (**C**) Elevation of ROS in PANC-1, MIAPaCa-2, and SW1990 72 h after PRX3 siRNA knockdown (×40). * *p* < 0.05; **** *p* < 0.0001 vs. negative control (NC).

PRX3 protein secretion from pancreatic PA cells PANC-1, RWP-1, SW1990, and MIAPaCa-2 was confirmed via Western blot analysis (Figure 4A and Figure S1). Elevated levels of the 26 kDa PRX3 protein were found in the cytosol and culture supernatant of PA cells.

#### 3.7.2. Correlation of PRX3 and CD44v9 Expression in PDAC

The relationship between PRX3 and the pancreatic cancer stem cell marker CD44v9 was investigated via double fluorescent IHC in Exp. 1 and confocal microscopy in the PANC-1, MIAPaCa2, SW1990, and RWP-1 cell lines (Figure 2C(g,h) and Figure 4B). Double staining and confocal microscopy revealed the elevation of both PRX3 and CD44v9 in the PA cell mitochondria and cell membrane and/or cytoplasm, respectively. An increase in CD44v9 was obvious in strongly PRX3-positive PDAC cells; nevertheless, no expression was found in cells with low PRX3 (Exp. 1). Interestingly, PRX3/CD44v9 double-positive PANC-1, MIAPaCa-2, and, less frequently, SW1990 cells with elevated CD44v9 in both the cell membrane and cytosol were often in the mitotic state.

#### 3.7.3. Effects of PRX3 Silencing on ROS Levels in PA Cells

A remarkable reduction in the protein level of PRX3 in PANC-1, MIAPaCa-2, RWP-1, and SW1990 cells transfected with PRX3 siRNA 2 (PRX3kn-2) compared with the siRNA control samples was demonstrated 72 and 96 h after the addition of siRNA to the cell culture (Figure S2). The results of the highly sensitive DCFH-DA-ROS assay demonstrated a significant elevation of intracellular ROS levels after PRX3 silencing with PRX3 siRNA-2 (Figure 4C). These results suggest that the antioxidant activity of PRX3 against ROS is part of the oxidative stress resistance mechanism in PDAC cell lines.

### 3.8. In Vivo Balb/c Nude Mice Xenograft Model

**Figure 5:**
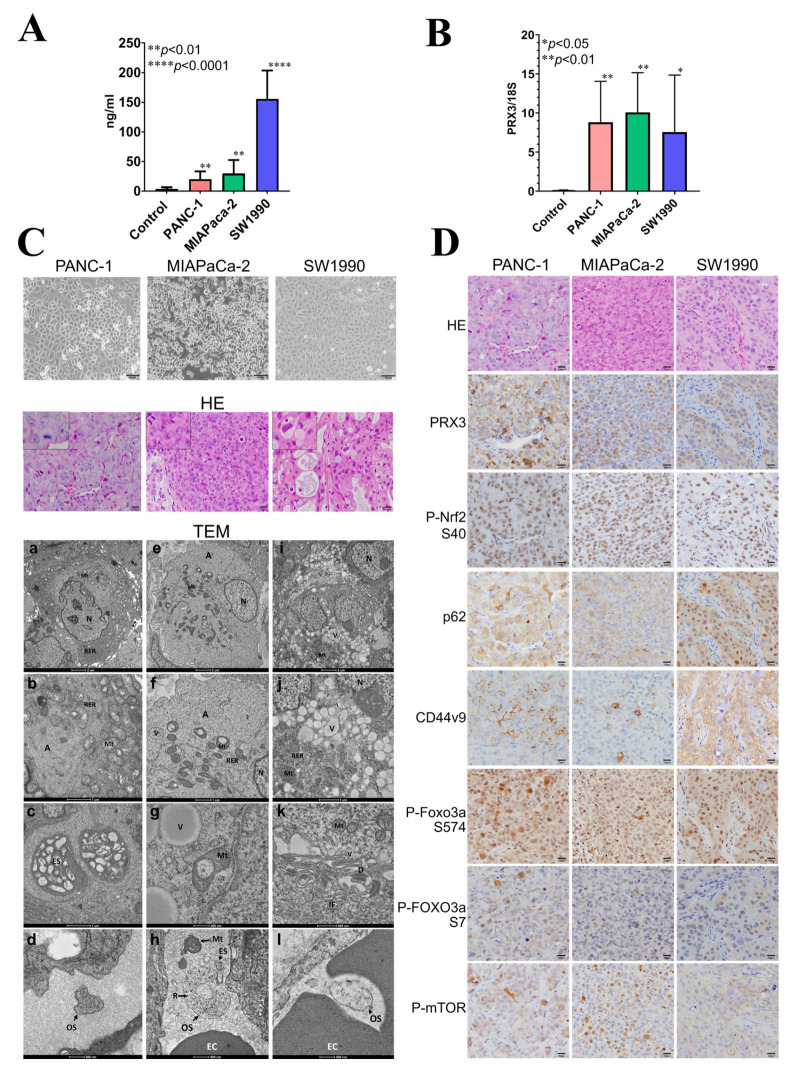
Tumor and blood analysis in the Balb/c nude mouse xenograft model. (**A**) Human PRX3 protein levels in nude mouse blood plasma detected via ELISA. ** *p* < 0.01; **** *p* < 0.0001. (**B**) EV PRX3 mRNA levels detected via RT-PCR. Note the significant increase in PRX3 protein and EV mRNA levels. The highest protein levels in the blood were found for SW1990 tumors. * *p* < 0.05; ** *p* < 0.01. (**C**) PANC-1, MIAPaCa-2, and SW1990 cells (×100); HE staining (×400) and transmission electron microscopic (TEM) analysis of tumors (×2000–×12,000). Note the mucus and intracellular vesicles in SW1990 cells and mitotic figures in PANC-1 and MIAPaCa-2. TEM analysis demonstrated an increased number of damaged mitochondria [Mt] with vesicles [V]; developed vacuoles in rough endoplasmic reticulum [RER]; actin cytoskeleton [A] and depots with exosomes in cancer cell lines (**a**–**c**;**e**–**g**;**i**–**k**); and vacuoles [V] (**j**), desmosomes [D], and intermediate filaments [IF] (**k**) in SW1990 cells. Exosomes [ES] and large oncosomes [OS] with ribosomes [R] were detected in the blood vessels with erythrocytes [EC] in all three examined PA cell lines (**d**,**h**,**l**). Furthermore, free mitochondria [Mt] were observed in the tumor blood vessels near the OS (**h**). (**D**) HE, PRX3, P-Nrf2, p62, CD44v9, P-FOXO3a-S574, P-FOXO3a-S7, and P-mTOR IHC in nude mouse tumors (×400). Note that the activation of Nrf2, FOXO3a, and mTOR is associated with PRX3 elevation.

The results obtained in the Balb/c nude mice xenograft model are presented in Figure 5.

To identify candidate blood biomarkers using the xenograft mouse model, PANC-1, MIAPaCa-2, and SW1990 cells were injected subcutaneously into Balb/c nude mice. PANC-1 and MIAPaCa-2 are the epithelial cell lines derived from the tumor tissue of patients with PA, while SW1990 originated from spleen metastasis from a patient with grade II PA. These cell lines were chosen because the in vitro growth characteristics mimic the aggressive clinical behavior of human PDAC; they have a fast growth rate, and they are known to form tumors readily in immune-compromised mice. PDAC is a heterogeneous disease with three major subtypes: classical, quasi-mesenchymal, and exocrine-like. Moreover, there is evidence for clinical outcome and therapeutic response differences between them.

The survival rate of Balb/c mice at the time of sacrifice was 100% in all groups. There was no particular effect on the general condition, food intake, water intake, or body weight of nude mice observed. We performed blood analyses and microscopic, pathological, electron microscopic, and immunohistochemical examinations of nude mice PANC-1, MIAPaCa-2, and SW1990 xenograft tumors. The tumors had metastasized to the lungs of nude mice.

#### 3.8.1. PRX3 Protein and EV mRNA Analyses in Nude Mice Blood Plasma

Firstly, the blood plasma of nude mice was subjected to ELISA using a Human PRX3 ELISA kit. A significant elevation of human PRX3 protein levels was detected for all examined PA cell lines (Figure 5A), with the highest increase observed for SW1990 cells. Next, nude mice blood plasma EVs (small exosomes and large oncosomes) were isolated from the nude mouse blood plasma samples using the exoRNeasy kit, the total EV RNA was extracted, and RNA quality was verified. The amount of isolated RNA was sufficient for performing reverse transcription and quantitative real-time (RT)-PCR analyses; therefore, the mRNA expression of human PRX3, Nrf2, p62, and CD44v was analyzed. As a result, a significant elevation of human PRX3 mRNA was found in the EVs released into the blood plasma of nude mice bearing tumors compared to the control (Figure 5B). However, no increase in human Nrf2, p62, and CD44v9 mRNA was observed in nude mice blood EVs.

#### 3.8.2. Histological, Electron Microscopic, and Immunohistochemical Analysis of Nude Mouse Tumors

Histopathological analyses demonstrated that PANC-1 and MIAPaCa-2 were very actively proliferating and contained frequent mitotic figures (Figure 5C). SW1990 tumors proliferated less but contained mucus inclusions and discolored vesicles in the cytoplasm of tumor cells, indicative of active exocytosis. Furthermore, within PA tumors, we observed the formation of numerous blood capillaries (Figure 5C).

Examination of PANC-1, MIAPaCa-2, and SW1990 tumors using TEM demonstrated well-developed rough endoplasmic reticulum (RER), actin cytoskeleton, increased mitochondria, and large depots with exosomes (ES) and numerous vesicles (Vs) in the cytoplasm and even inside the mitochondria (Mt) (Figure 5C). PANC-1 and MIAPaCa-2 had similarities in terms of ultrastructure but differed from SW1190, which contained a large number of vacuoles, desmosomes, and intermediate filaments in the cytoplasm (Figure 5C).

Interestingly, in the blood vessels of PANC-1, MIAPaCa-2, and SW1990 tumors, numerous EVs, exosomes (ES) (60–100 nm), and large oncosomes (500–1000 nm) with ribosomes were observed (Figure 5C). Furthermore, unexpectedly, free mitochondria were also detected inside the blood vessels near the large oncosomes (OSs), mostly in MIAPaCa-2 and PANC-1 cells. This may help to suggest that tumor cells are capable of releasing not only EVs but also mitochondria directly into the blood vessel. Further investigation is required to confirm this finding.

The expression of PRX3, P-Nrf2, p62, CD44v9, P-FOXO3a S574, S7, and P-mTOR was investigated and compared using IHC in the serial sections of nude mice tumors (Figure 5D). The overexpression of PRX3 in PANC-1, MIAPaCa-2, and SW1990 tumors was correlated with that of P-Nrf2, p62, CD44v9, P-FOXO3a S574, S7, and P-mTOR, thus indicating co-operative activation of oxidative stress-related signaling, including Prx, Nrf2, CD44v9, FOXO3a, and mTOR pathways in PDAC. From the IHC results, CD44v9 expression is mostly membranous in PANC-1 and MIAPaCa-2 cells but cytoplasmic in SW1990 cells. In addition, FOXO3a phosphorylation was found to be mainly due to JNK and p38/ERK MAP kinase activities.

### 3.9. PRX3 Protein and EV mRNA as Novel Potential Biomarkers of Human PDAC (Exp. 2)

**Figure 6:**
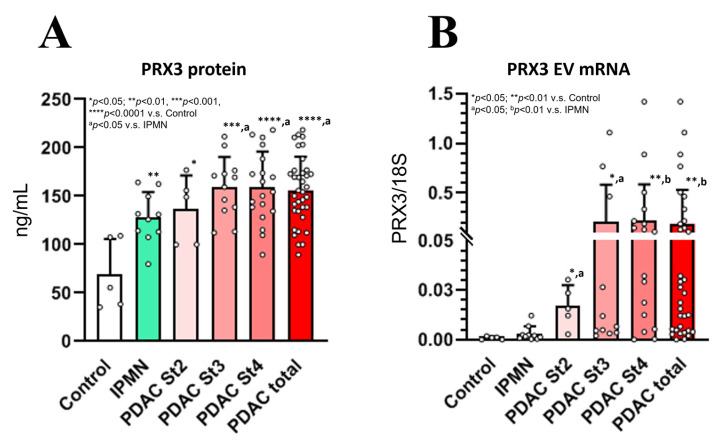
PRX3 protein (**A**) and EV mRNA levels (**B**) in the blood serum of PDAC, IPMN, and healthy control patients (Exp. 2). * *p* < 0.05; ** *p* < 0.01; *** *p* < 0.001; **** *p* < 0.0001 vs. Control group. ^a^ *p* < 0.05; ^b^ *p* < 0.01 vs. IPMN group.

The patient characteristics at baseline are presented in Table S1. PRX3 protein and EV mRNA levels were measured via PRX3 ELISA and quantitative RT-PCR analyses at the time of PDAC and IPMN diagnosis, and the results are presented in Figure 6. ROC curves were generated from the data, and the cut-off values were selected (PDAC vs. IPMN + Control) (Figure S3).

#### 3.9.1. Analysis of Blood Serum PRX3 Protein Levels Using ELISA and Correlation Analysis

PRX3 protein levels were significantly elevated in the blood serum of PDAC stage 2–4 patients (Figure 6A). Furthermore, a less prominent but significant increase in PRX3 serum protein levels was found in IPMN patients with respect to the control. The PRX3 protein levels of stage 3 and 4 PDAC patients were significantly higher than those of the IPMN group. The median baseline PRX3 protein serum levels in stage 2–4 PDAC patients were 157 ng/mL (range: 89–218 ng/mL) (*p* < 0.0001 vs. control and *p* < 0.05 vs. IPMN), while healthy subjects had a median of 73 ng/mL (range: 35–140 ng/mL), and IPMN subjects had PRX3 protein median serum levels of 136 ng/mL (range: 82–205 ng/mL) (*p* < 0.01 vs. control) (Figure 6A). The cut-off values were detected via median analysis and the Yunden index.

The cut-off value for the PRX3 protein was set at 140 ng/mL. The analysis of PRX3 showed that 26 PDAC patients were positive for this biomarker, while no controls and 3 IPMN patients showed PRX3 protein serum levels above the selected cut-off value. ROC curve analysis of the PRX3 protein showed an AUC value of 0.95 for the differentiation between total PDAC patients and healthy control samples (Table S6). In the receiver operating characteristic curve analysis, the overall sensitivity and specificity of serum PRX3 protein levels were, respectively, 72.2% and 80.0% for differentiating PDAC patients from IPMN combined with healthy control patients (Yunden index 0.483) and 75% and 80% (Yunden index 0.694) for differentiating PDAC patients from healthy controls, with a 140 ng/mL cut-off value (Table S6). In IPMN patients, adding PRX3 protein levels to the analysis increased the sensitivity and specificity of the CA19-9, DUPAN-2, and Span-1 levels, which were not increased in the IPMN group.

High and low blood serum PRX3 protein levels detected via ELISA showed significant positive correlations with PDAC diagnosis (*p* = 0.012), extrapancreatic nerve plexus invasion (pPL) (positive vs. negative, *p* = 0.045), gender (male vs. female, *p* = 0.023), diabetes (positive vs. negative, *p* = 0.001), lipase (>35 U/L vs. 0–35 U/L, *p* = 0.025), gamma glutamyl transferase (γGT) (>60 U/L vs. 5–60 U/L, *p* = 0.009), alkaline phosphatase (ALP) (>360 vs. 115–359 IU/L, *p* = 0.004), and alanine transaminase (ALT) (>28 IU/L vs. 6–27 IU/L, *p* = 0.022). A positive correlation trend with lymph node metastasis (pN factor) (N1,2 vs. N0, *p* = 0.093) and Span-1 levels (>30 U/mL vs. 0–30 U/mL, *p* = 0.073) (Table 3) was observed.

Significantly elevated levels of circulating EV PRX3 mRNA were detected in PDAC patients compared to healthy controls and IPMN subjects (Figure 6B). The cut-off value for PRX3 EV mRNA was set as 0.01 PRX3/18S. PRX3 EV mRNA was positive in 23 PDAC patients, while no controls and only 1 IPMN patient had PRX3 EV mRNA serum levels above the selected cut-off value, with AUC values of 0.85 for the differentiation between PDAC and IPMN combined with healthy control patients (sensitivity: 61.1%; specificity: 100.0%; Yunden index: 0.611) and 0.82 (sensitivity: 63.9%; specificity: 100.0%; Yunden index: 0.639) for the differentiation from only the controls (Table S6). The median baseline EV PRX3 mRNA serum levels were 0.18 PRX3/18S (range: 0.0002–1.4186 PRX3/18S) in PDAC stage 2–4 patients (*p* < 0.01 vs. control and *p* < 0.01 vs. IPMN), while healthy subjects had a median of 0.0008 PRX3/18S (range: 0.0000–0.0018 PRX3/18S), and IPMN subjects had a median of 0.003 PRX3/18S PRX3 EV mRNA serum levels (range: 0.0004–0.0122 PRX3/18S) (*p* < 0.01 vs. control) (Figure 6B).

The increase in EV PRX3 mRNA was significantly associated with PDAC diagnosis (PDAC vs. IPMN + control, *p* = 0.001); tumor size (>2 cm vs. ≤2 cm, *p* = 0.012); pN status (N1,2 vs. N0, *p* = 0.004); PDAC infiltrative growth pattern (INFc vs. INFab, *p* = 0.048); and increased levels of CA19-9 (>37 U/mL vs. 0–37 U/mL, *p* = 0.043), Span-1 (>30 U/mL vs. 0–30 U/mL, *p* = 0.010), CEA (>5.0 ng/mL vs. 0–5.0 ng/mL, *p* = 0.037), amylase (>126 U/L vs. 40–126 U/L, *p* = 0.030), lipase (>35 U/L vs. 0–35 U/L, *p* = 0.025), ALP (>360 vs. 115–359 IU/L, *p* = 0.004), AST (>34 IU/L vs. 13–33 IU/L, *p* = 0.043), and ALT (>28 IU/L vs. 6–27 IU/L, *p* = 0.025) (Table 3). However, no significant associations were found between PRX3 EV mRNA levels in the blood serum and patients’ age, gender, drinking, smoking status, body mass index (BMI), or past history of diabetes (Table 3).

#### 3.9.2. Prognosis for Elevated Blood PRX3 Protein and EV mRNA (Exp. 2)

The univariate analyses of survival correlations with clinico-pathological variables and blood serum PRX3 protein and EV mRNA levels are presented in Table S7. According to the results of the univariate analysis, poor patient survival was significantly associated with PRX3 EV mRNA (high vs. low; cut-off 0.01 PRX3/18S; HR 5.123, *p* = 0.010); pN status (N1,2 vs. N0; HR 11.143, *p* = 0.020), tumor differentiation (poorly vs. well and moderately differentiated; HR 4.234, *p* = 0.004); higher T category (T4 vs. T1,2,3; HR 3.010, *p* = 0.003); M status (M1 vs. M0; HR 7.283, *p* = 0.000); clinico-pathological stage (stage 4 vs. 1,2,3; HR 3.589, *p* = 0.009); tumor infiltrative growth pattern (INFc vs. INFab; HR 4.555, *p* = 0.004); and tumor size (>4 cm vs. ≤4 cm; HR 5.561, *p* = 0.000) (Table S7). Univariate analysis showed no significant correlation with survival for CA19-9, CEA, DUPAN-2, Span-1, or PRX3 protein levels in the blood serum.

Multivariate Cox proportional hazards regression analysis revealed a significant correlation between PRX3 EV mRNA (high vs. low; HR cut-off 0.01 PRX3/18S) and pN status (N1,2 vs. N0; HR 0.22, *p* = 0.011) and tumor differentiation (poorly vs. well and moderately differentiated; HR 0.215, *p* = 0.025). According to these results, the elevation of PRX3 EV mRNA, but not the PRX3 protein, in the blood serum was strongly correlated with poor PDAC patient survival and correlated with the pN status and poor tumor differentiation.

## 4. Discussion

The present study demonstrated that the PRX3 protein and EV mRNA are potential novel biomarker candidates useful for the early detection, diagnosis, and prognosis of human invasive PDAC. PRX3 was selected and investigated based on the results of proteomic and bioinformatic analyses, demonstrating strong and frequent elevations in PDAC in correlation with the activation of oxidative stress resistance mechanisms via Nrf2 and FOXO3a. From the results of immunohistochemical examination, granular staining of cytoplasmic PRX3 was observed in the PA cells of most specimens, regardless of staining intensity. Importantly, PRX3 protein and EV mRNA levels in the blood of PDAC stage 2–4 patients were significantly elevated and associated with PDAC development. Moreover, the release of PRX3 EV mRNA into the blood was significantly associated with the poor survival of PDAC patients and correlated with pN status and tumor size. The elevation of PRX3 protein levels was further observed in the blood serum of IPMN patients, which is considered a precancerous condition and is characterized by growth within the pancreatic ducts, mucinous epithelial cells, and genetic alterations in KRAS and guanine nucleotide-binding protein and alpha-stimulating activity polypeptide (GNAS) [20].

Recent research reported that mitochondria are involved in oncogenesis and the alteration of cancer cell metabolism, and abnormal mitochondria are the source of a large amount of ROS produced in cancer cells. The Txn-Prx system is an important cellular defense system against oxidative stress, and it is reported as a potential target for cancer therapy; moreover, cancer cell mitochondria contain high levels of PRX3, which is a member of the Prx family and a thioredoxin-dependent hydrogen peroxide scavenging enzyme [21].

PRX3 is important for maintaining mitochondrial integrity and plays a major role in mitochondrial redox signaling and oxidative stress resistance mechanisms; it protects cancer cells from large amounts of reactive oxygen species (ROS) produced by abnormal mitochondria [22]. Other protein members of the Txn-Prx family—including PRX1, 2, 4, 5, and 6, which are localized in the cytosol, endoplasmic reticulum, or peroxisomes—were not significantly overexpressed in PDAC. Furthermore, in this study, proteomic analyses demonstrated that the expression of superoxide dismutase 2 and 3 and catalase was inhibited in PDAC compared to surrounding pancreatic tissue, indicating that oxidative stress resistance mechanisms involving the Txn-Prx system in mitochondria are specific for PDAC. In addition, the mutated KRAS gene, which is strongly overexpressed in pancreatic cancer cells, has been reported to induce ROS production. In addition, the ROS-induced activation of transcriptional factor Nrf2 has been reported to regulate the expression of the PRX family [23,24,25]. PRX3 was also shown to be activated by sensitive oxidative stress indicators—FOXO3a, NF-kB, and SP1—in H9c2 cardiac cells and the hippocampus [26,27,28]. The expression of PRX3 is increased in prostate, breast cancer, and hepatocellular carcinoma [24,29,30,31]. ROS and their important source, hydrogen peroxide, are further known to activate transcriptional factors Nrf2 and NF-κB and MAP kinases p38, ERK1/2, JNK, and AKT, thus promoting cell proliferation and anti-apoptotic effects [32]. Therefore, the cause of the significant elevation of PRX3 in PA cells is the induction of oxidative stress resistance mechanisms in cancer cells and the elimination of ROS for cancer cell survival. In support of the importance of PRX3 for ROS elimination and PA cell survival, our results indicated that the knockdown of PRX3 in PDAC-1, MIAPaCa-2, and SW1990 PA cells induced an increase in ROS in the cytoplasm of PA cells. The PRX3-related PA cell mechanisms involve the activation of actin signaling, the development of EPR and damaged mitochondria, and the formation and release of EVs of differential sizes, such as exosomes and oncosomes.

In exploring whether PRX3 may become a PDAC biomarker, we detected PRX3 expression in the culture supernatant of pancreatic cancer cell lines. The fact that PRX3 protein is secreted from PA cells suggests that it can also be released from tumor cells into the blood. Next, it was observed that human PRX3 protein was released from the PANC-1, MIA-PaCa-2, and SW1990 tumors into nude mice blood. When mouse tumors were subjected to electron microscopic analysis, exosomes and oncosomes with ribosomes, as well as free mitochondria, were found in the blood vessels in the tumors. This supported the idea that not only PRX3 protein but also its mRNA in EVs can be released into the blood from PA cells, which may become a novel marker for PDAC diagnosis.

To our knowledge, the marked elevation of PRX3 EV mRNA and proteins in the blood serum of PDAC and IPMN patients is a novel, important finding of the present study that can be applied to blood diagnosis. The cut-off value for PRX3 protein was set to 140 ng/mL, and the cut-off for PRX3 EV mRNA was set to 0.01 PRX3/18S. The analysis of both PRX3 protein and EV mRNA markers together may facilitate more efficient diagnoses. In PDAC patients at stages 2–4, the sensitivity and specificity of PRX3 were increased over CEA, DUPAN-2, and Span-1 but not CA19-9 (Table S6). On the other hand, being elevated in IPMN, PRX3 protein may become a biomarker for the early detection of IPMN and PDAC. No increase in CA19-9, CEA, DUPAN-2, or Span-1 was detected in the IPMN group. Furthermore, from our results, the combination of PRX3 with CA-19-9, DUPAN-2, and Span-1 was synergistic, and if combined with existing biomarkers for early detection, adding PRX3 protein and EV mRNA levels to the analysis increased their sensitivity and specificity.

The isolation of EVs, which are not easily destroyed in the blood serum, appeared to be a limiting step for sufficient RNA extraction and the RT-PCR analysis of PRX3 mRNA expression. Here, we observed ribosomes in EVs and the depots with exosomes using TEM imaging of tumors; however, it is still unclear how specific mRNAs are being packaged into exosomes/oncosomes. In a previous study, elevated blood serum PRX3 protein was reported as a useful diagnostic and/or prognostic predictor in patients with hepatocellular carcinoma; however, EV markers were not analyzed [33].

Recently, cancer researchers have dedicated great effort to understanding the impacts of EVs on intercellular communication and their mediation of different aspects of cancer growth, intending to develop novel approaches to fight the disease. Currently, EVs are thought to comprise different types of unique particles, such as exosomes, other small exosome-sized EVs, microvesicles (MVs), arrestin domain-containing protein 1-mediated microvesicles (ARMMs), apoptotic bodies, and oncosomes. Exosomes are small extracellular vesicles (sEVs) that play a crucial role in intercellular communication in physiological and pathological processes [34]. The high metastatic potential of tumors has been linked to markedly high EV release; for instance, in melanoma, it exerts a crucial role in rapid tumor progression. PDAC tumors were reported to be capable of establishing a co-operation network mediated by EVs, which is controlled by CSC and agrin via transcriptional regulator (YAP) activation, which allowed tumors to adapt and thrive [35]. The mesenchymal nature and the larger percentage of CSCs in the tumor drive the release of EVs [36]. Furthermore, EVs were reported to be capable of inducing tumor-favorable phenotypes in recipient cells and metastatic sites. Thus, the re-education of mesenchymal stromal cells, fibroblasts, and endothelial cells may promote tumor growth, angiogenesis, and metastasis [36]. In our study, numerous small and large EVs were released into the tumor blood vessels of nude mice bearing tumors, and those tumors metastasized to the lungs.

Additionally, the diagnostic and therapeutic use of EVs secreted by various cell types, mainly CSCs and tumor-initiating progenitor cells, is believed to have significant advantages over the use of parent cells [37]. In this study, we found a correlation between PRX3 and the cancer stem cell membranous marker CD44v9, suggesting that actively metastasizing CD44v9-positive CSCs can release PRX3 into the blood, which may assist in their detection and diagnosis. Furthermore, the clinico-pathological analysis demonstrated that high levels of CD44v9 were associated with high levels of PRX3 in PDAC patients. Interestingly, CD44v9 in the cytoplasm of PA cells drove mitosis. Previously, in line with our results, CD44v9 was elevated during the mitotic phases of the cell cycle and was suggested to be associated with cell proliferation in pancreatic cancer cells (PCCs) with an epithelial phenotype [38]. Thus, Kiuchi et al. demonstrated the high surface and cytoplasmic expression of CD44v9 in mitotic stages (prophase, metaphase, anaphase, telophase, and cytokinesis), and its low expression in interphase was demonstrated in all examined mesenchymal phenotype PA cell lines. In addition, in line with our results, the Nrf2/FOXM1/PRX3 mitochondrial pathway was activated, and it helped colon and endometrial CSC survival [22,39,40].

IHC analyses in human PDAC tissue further demonstrated that PRX3 protein levels were high in lesions corresponding to PanIN. There are currently no reports on the expression of PRX3 in PanIN, and this result suggests that PRX3 could become a useful biomarker for detecting precancerous lesions. However, since the number of PanIN cases examined in this study was small, it is necessary to examine this possibility in detail by targeting more cases.

Regarding the relationship between post-operative survival time and PRX3, no difference was observed in the survival curves regarding overall survival time; in the 1-year survival curve, a high number of PRX3-positive cases had a poor prognosis. This suggests that PRX3 could become a short-term prognostic predictor. As the 5-year survival rate after surgery for pancreatic cancer patients is approximately 8%, and it is known that the survival rate deteriorates rapidly to around 70% in a short period within 1 year after surgery, the PRX3 level in PDAC may be of short-term prognostic significance. In addition, it was detected that the analysis of PRX3 expression in PDAC could be an important prognostic factor in patients with elevated SPan-1 and DUPAN-2, two existing PDAC biomarkers. It has been reported that SPan-1 can be used as a post-operative prognostic predictor and that the combined evaluation of tumor diameter via CT and the measurement of CA19-9 levels could be applied to predict the early recurrence of pancreatic cancer [18,41]. Based on these results, it could be suggested that PRX3 in PDAC tissues may be more useful when used in short-term prognostic prediction or as an accurate prognostic auxiliary factor applied in combination with existing tumor markers. In contrast to our results, in another study, when the expression of PRX3 was confirmed via immunohistochemistry in 69 cases of pancreatic cancer, no significant association with overall survival was observed [42].

In correlation analysis, increased blood serum PRX3 EV mRNA and protein levels, as well as PRX3 protein in the PDAC tissue, were associated with invasive tumor-mode infiltrative growth pattern INFc and lymph node metastasis, suggesting that PRX3 EV mRNA-containing oncosomes and proteins may drive tumor cell infiltration, invasion, and metastasis. Furthermore, PRX3 IHC staining in PDAC demonstrated strong positive expression at the site of invasion, suggesting its influence on tumor cell invasion ability. In recent studies, PRX3 has been reported to be associated with the proliferative ability of breast cancer and hepatocellular carcinoma, and it was correlated with Ki67 staining in cervical cancer [43,44,45]. In contrast to these results, we did not observe a clear relationship between PRX3 staining and the Ki67 index in PDAC.

A significant association between PRX3 EV mRNA elevation in the blood serum and poor PDAC patient survival was found. In addition, PRX3 could be useful as a prognostic marker under certain conditions. The above results revealed that PRX3 is overexpressed in invasive PDAC and precancerous lesions, and its protein and EV mRNA are secreted into the blood; therefore, they may be applied in clinical practice as early diagnostic PDAC markers. PRX3 can also be suggested as a potential therapeutic target.

Although the present study provides important findings on the novel early PDAC biomarker PRX3 protein and its EV mRNA, it has several limitations. First, the number of PDAC, IPMN, and healthy control patients subjected to blood analysis in Exp. 2 was not high, and there were no external validation sets. Second, further studies are required to validate the relevance of PDAC-derived EVs (exosomes and oncosomes) and PRX3 mRNA in pancreatic carcinogenesis. The elevation of PRX3 protein levels could be less specific than EV mRNA levels. The fact that mitochondria were found inside the blood vessels of PDAC tumors via TEM analysis should be further verified, and whether PRX3 could also be released within the mitochondria should be researched. Future studies are needed to investigate the mechanism of action and the therapeutic potential of targeting PDAC-derived exosomes to understand their involvement in the metastasis process.

## 5. Conclusions

Our results show that both PRX3 protein and EV mRNA were significantly elevated in the blood serum of PDAC patients, suggesting their potential as novel blood biomarkers in the diagnosis of human invasive PDAC. Still, there is a possibility that PRX3 protein elevation reflects a response to the formation of broad oxidative stress in the tumor; however, the strong ability of PA cells to secrete PRX3 protein and its EV mRNA into the blood may be very useful for early PDAC diagnosis. Furthermore, PRX3 is likely to be released by PDAC CSCs, which may help in their diagnosis. Moreover, PRX3 can become a prognostic factor for PDAC in the short post-operative period, and the combination of PRX3 with SPan-1 and DUPAN-2 is expected to improve the accuracy of PDAC prognosis. Mechanistically, PRX3 overexpression was correlated with the activation of P-Nrf2/p62 and P-FOXO3a Ser594/S7 signaling in human PDAC.

## Figures and Tables

**Table 1:** Altered upstream regulators and their function, type, and activation z-score in PDAC detected via proteomic analysis and Ingenuity Pathway Analysis.

Upstream Regulator	Function	Molecule Type	Activation z-Score	*p*-Value of Overlap
Tgf-β	Cell proliferation, fibrogenesis	group	3.421	6.43 × 10^−7^
SMAD2	Cell proliferation, fibrogenesis	TR	2.415	7.3 × 10^−4^
SMAD3	Cell proliferation, fibrogenesis	TR	2.784	3.1 × 10^−8^
SMAD4	Cell proliferation, fibrogenesis	TR	2.284	2.03 × 10^−5^
NFkB	Cell growth, proliferation	complex	3.604	1.79 × 10^−4^
Nrf2 (NFE2L2)	Oxidative stress resistance	TR	3.229	0.00741
CD44	Stem cell marker, cell adhesion	other	3.077	1.48 × 10^−5^
CTNNB1	Cell growth, proliferation	TR	2.975	7.32 × 10^−9^
SP1	Cell growth, proliferation	TR	3.067	0.00125
HIF1A	Cell growth, proliferation	TR	2.784	0.00306
FOXA2	Cell proliferation, stress resistance	TR	2.240	1.92 × 10^−5^
FOXO3	Cell proliferation, mtDNA transcription	TR	2.390	0.0404
P38	Cell growth, proliferation	group	2.785	0.0491
AKT1	Cell growth, proliferation	kinase	2.007	0.00155
PI3K	Cell growth, proliferation	complex	2.607	0.0355
ERK1/2	Cell growth, proliferation	group	2.592	9.99 × 10^−6^
SMARCA4	Cell proliferation, DNA repair	kinase	3.251	6.88 × 10^−10^
IGF1R	Cell growth, proliferation	TMR	2.186	4.75 × 10^−4^
IGF2BP1	Cell growth, proliferation	TLR	2.646	1.82 × 10^−7^
STAT1	Cell survival, apoptosis suppression	TR	2.017	0.00745
STAT4	Cell survival, apoptosis suppression	TR	2.800	0.0391
YAP1	EMT, actin cytoskeleton	TR	3.179	0.00181
VEGFA	Angiogenesis, fibrogenesis	growth factor	2.18	0.0398
FGF2	Angiogenesis, fibrogenesis	growth factor	2.653	2.65 × 10^−5^
TEAD1	Angiogenesis, actin cytoskeleton	TR	2.236	0.00211
TEAD4	Angiogenesis, actin cytoskeleton	TR	2.449	0.0023
OSM	Inflammation	cytokine	3.194	3.03 × 10^−4^
IL4	Inflammation	cytokine	3.75	1.27 × 10^−10^
IL5	Inflammation	cytokine	3.239	0.00115
IL6	Inflammation	cytokine	3.217	2.81 × 10^−4^
Alpha catenin	Cell adhesion	group	−3.81	3.81 × 10^−11^

Abbreviations: TR, transcription regulator; TLR, translation regulator; TMR, transmembrane receptor; EMT, epithelial-mesenchymal transition.

**Table 2:** PRX3 expression in PDAC and its association with clinico-pathological variables of PDAC patients (Exp. 1).

Clinico-Pathological Variables	Number of Patients (%)					
	Total	High		Low		*p*
Age(y) ≤70	50	31	(62.0%)	19	(38.0%)	0.529
>70	50	34	(68.0%)	16	(32.0%)	
Gender Male	47	30	(63.8%)	17	(36.2%)	0.817
Female	53	35	(66.0%)	18	(34.0%)	
CA19-9 0–37 U/mL	24	15	(62.5%)	9	(37.5%)	0.768
>37 U/mL	76	50	(65.8%)	26	(34.2%)	
CEA 0–5.0 ng/mL	68	46	(67.6%)	22	(32.4%)	0.553
>5.0 ng/mL	23	14	(60.9%)	9	(39.1%)	
DUPAN-2 0–150 U/mL	43	30	(69.8%)	13	(30.2%)	0.173
>150 U/mL	33	18	(54.5%)	15	(45.5%)	
SPan-1 0–30 U/mL	36	23	(63.9%)	13	(36.1%)	0.959
>30 U/mL	59	38	(64.4%)	21	(35.6%)	
CD44v9 Negative	51	29	(56.9%)	22	(43.1%)	**0.044**
Positive	49	36	(73.5%)	13	(26.5%)	
P-Nrf2 (S40) Low	44	20	(45.5%)	24	(54.5%)	**0.003**
High	56	45	(80.4%)	11	(19.6%)	
FOXO3a Low	62	31	(50%)	31	(50%)	**0.0001**
High	38	34	(89.5%)	4	(10.5%)	
Ki67 index Low	50	32	(64.0%)	18	(36.0%)	0.834
High	50	33	(66.0%)	17	(34.0%)	
Tumor size ≤2cm	23	16	(69.6%)	7	(30.4%)	0.601
>2cm	77	49	(63.6%)	28	(36.4%)	
Differentiation Well, Mod	91	58	(63.7%)	33	(36.3%)	0.488 *
Poor	9	7	(77.8%)	2	(22.2%)	
Infiltration growth pattern INFa,b	87	53	(60.9%)	34	(39.1%)	**0.013**
INFc	10	10	(100.0%)	0	(0.0%)	
Lymphatic invasion (ly) Negative	35	29	(29.0%)	6	(6.0%)	0.159
Positive	65	48	(48.0%)	17	(17.0%)	
Venous invasion (v) Negative Positive	62 38	38 27	(38.0%) (27.0%)	24 11	(24.0%) (11.0%)	0.169
T pT1,2	80	54	(67.5%)	26	(32.5%)	0.295
pT3,4	20	11	(55.0%)	9	(45.0%)	
N Negative	53	34	(64.2%)	19	(35.8%)	0.850
Positive	47	31	(66.0%)	16	(34.0%)	
M Negative	99	64	(64.6%)	35	(35.4%)	1.000 *
Positive	1	1	(100.0%)	0	(0.0%)	
Stage 1	41	29	(70.7%)	12	(29.3%)	0.316
2,3,4	59	36	(61.0%)	23	(39.0%)	

Pearson Chi-square or Fisher (*) test. IHC scores from 1 to 4 were weakly positive (Low), and those from 5 to 9 were strongly positive (High). The dichotomization was performed using the median split method. *p* values for significant findings are shown in bold.

**Table 3:** Correlation between PRX3 protein and EV mRNA levels in the blood serum and clinico-pathological variables (Exp. 2).

	PRX3 Protein (>140 ng/mL)				PRX3 EV mRNA (>0.01 PRX3/18S)			
Factors	Total	High	Low	*p*	Total	High	Low	*p*
All patients	51				51			
Age ≤70 >70	25 26	15(60.0%) 13(50.0%)	10(40.0%) 13(50.0%)	0.569	25 26	10(40.0%) 12(46.2%)	15(60.0%) 14(53.8%)	0.436
Gender Male Female	20 31	15(75.0%) 13(41.9%)	5(25.0%) 18(58.1%)	**0.023**	20 31	9(45.0%) 13(41.9%)	11(55.0%) 18(58.1%)	0.528
Drinking Non-drinker Drinker	18 33	11(61.1%) 17(51.5%)	7(38.9%) 16(48.5%)	0.606	18 33	9(50.0%) 13(39.4%)	9(50.0%) 20(60.6%)	0.331
Smoking Non-smoker Smoker	30 21	15(50.0%) 13(61.9%)	15(50.0%) 8(38.1%)	0.189	30 21	12(40.0%) 10(47.6%)	18(60.0%) 11(52.4%)	0.342
BMI <25 ≥25	38 13	18(47.4%) 4(30.8%)	20(52.6) 9(69.2%)	0.238	38 13	18(47.4%) 10(76.9%)	20(52.6%) 3(23.1%)	0.146
Diabetes Negative Positive	21 30	7(33.3%) 21(70.0%)	14(66.7%) 9(30.0%)	**0.001**	21 30	9(42.9%) 13(43.3%)	12(57.1%) 17(56.7%)	0.601
Diagnosis IPMN, Control PDAC	15 36	3(20.0%) 25(69.4%)	12(80.0%) 11(30.6%)	**0.012**	15 36	1(6.7%) 21(58.3%)	14(93.3%) 15(41.7%)	**0.001**
**Blood analysis in PDAC, IPMN and Control patients**								
CA19-9 0–37 U/mL >37 U/mL	22 29	10(45.5%) 18(62.1%)	12(54.5%) 11(37.9%)	0.306	22 29	6(27.3%) 16(55.2%)	16(72.7%) 13(44.8%)	**0.043**
Span-1 0–30 U/mL >30 U/mL	19 17	7(36.8%) 12(70.6%)	12(63.2%) 5(29.4%)	0.073	19 17	4(21.1%) 11(64.7%)	15(78.9%) 6(35.3%)	**0.010**
DUPAN-2 0–25 U/mL >25 U/mL	14 13	4(28.6%) 8(61.5%)	10(71.4%) 5(38.5%)	0.267	14 13	3(21.4%) 7(53.8%)	11(78.6%) 6(46.2%)	0.089
CEA 0–5.0 ng/mL >5.0 ng/mL	27 24	13(48.1%) 15(62.5%)	14(51.9%) 9(37.5%)	0.521	27 24	8(29.6%) 14(58.3%)	19(70.4%) 10(41.7%)	**0.037**
Amylase 40–126 U/L >126 U/L	36 15	17(47.2%) 11(73.3%)	19(52.8%) 4(26.7%)	0.193	36 15	12(33.3%) 10(66.7%)	24(66.7%) 5(33.3%)	**0.030**
Lipase 0–35 U/L >35 U/L	9 17	1(11.1%) 11(64.7)	8(88.9%) 6(35.3%)	**0.025**	9 17	1(11.1%) 11(64.7%)	8(88.9%) 6(35.3%)	**0.025**
γGT 5–60 U/L >60 U/L	24 27	8(33.3%) 20(74.1%)	16(66.7%) 7(25.9%)	**0.009**	24 27	7(29.2%) 15(55.6%)	17(70.8%) 12(44.4%)	0.052
ALP 115–359 IU/L >360 IU/L	28 23	11(39.3%) 17(73.9%)	17(60.7%) 6(26.1%)	**0.004**	28 23	7(25.0%) 15(65.2%)	21(75.0%) 8(34.8%)	**0.004**
AST 13–33 IU/L >34 IU/L	22 29	10(45.5%) 18(62.1%)	12(54.5%) 11(37.9%)	0.139	22 29	6(27.3%) 16(55.2%)	16(72.2%) 13(44.8%)	**0.043**
ALT 6–27 IU/L >28 IU/L	23 28	9(39.1%) 19(67.9%)	14(60.9%) 9(32.1%)	**0.022**	23 28	6(26.1%) 16(57.1%)	17(73.9%) 12(42.9%)	**0.025**
**Tumor analysis**								
Tumor size ≤2 cm >2 cm	4 32	3(75.0%) 22(68.8%)	1(25.0%) 10(31.2%)	0.644	4 32	0(0%) 21(65.6%)	4(100.0%) 11(34.4%)	**0.012**
T category T2, 3 T4	15 21	10(66.7%) 15(71.4%)	5(33.3%) 6(28.6%)	0.602	15 21	10(66.7%) 11(52.4%)	5(33.3%) 10(47.6%)	0.391
N N0 N1,2	11 25	5(45.5%) 16(64.0%)	6(54.5%) 9(36.0%)	0.093	11 25	9(81.8%) 16(64.0%)	2(18.2%) 9(36.0%)	**0.004**
M M0 M1	22 14	15(68.2) 10(71.4%)	7(31.8%) 4(28.6%)	0.497	22 14	11(50.0%) 10(71.4%)	11(50.0%) 4(28.6%)	0.178
Inf. growth pattern INFab INFc	19 17	13(68.4%) 12(70.6%)	6(31.6%) 5(29.4%)	0.564	19 17	14(73.7%) 7(41.2%)	5(27.3%) 10(58.8%)	**0.048**
Stage ^‡^ 2 3, 4	5 31	3(60.0%) 22(71.0%)	2(40.0%) 9(29.0%)	0.564	5 31	3(60.0%) 18(58.1%)	2(40.0%) 13(41.9%)	0.389
Differentiation ^†^ Well and Moderate Poor	19 17	13(68.4%) 12(70.6%)	6(31.6%) 5(29.4%)	0.436	19 17	9(47.4%) 12(70.6%)	10(52.6%) 5(29.4%)	0.142
pPL Negative Positive	4 14	2(50.0%) 10(71.4%)	2(50.0%) 4(28.6%)	**0.045**	4 14	1(25.0%) 9(64.3%)	3(75.0%) 5(35.7%)	0.389

Pearson’s Chi-square test; pT factor: pathological T factor; pM factor: pathological M factor; pPL, extrapancreatic nerve plexus invasion; γGT, gamma glutamyl transferase; ALP, alkaline phosphatase; AST, aspartate aminotransferase, ALT, alanine transaminase; ^†^ Well and Moderately vs. Poorly differentiated; ^‡^ Stage 2 vs. Stages 3 and 4. *p* values for significant changes are shown in bold.

## Data Availability

Data are contained within the article.

## Supplementary Materials

The following supporting information can be downloaded at https://www.mdpi.com/article/10.3390/cancers17132212/s1, Figure S1: Secretion of PRX3 in the culture medium of PA cell lines (Western blot). Figure S2: In vitro PRX3 silencing using siRNA (Western blot) in PANC-1, MIAPaCa-2, and SW1990 cells. Figure S3. ROC curves for PRX3 protein and EV mRNA levels in the PDAC, IPMN, and healthy control patients. Table S1. Clinicopathological characteristics of PDAC and IPMN patients (Exp. 1 and 2). Table S2. Proteins up-regulated more than 5-fold in human PDAC detected by LC-MS/MS analysis. Table S3. Biomarker Filter Analysis by IPA. Table S4. Canonical Pathway analysis by IPA. Table S5. Univariate and multivariate analyses in PDAC patients in respect to survival (Exp. 1). Table S6. Sensitivity, Specificity and AUC values for PRX3 protein, PRX3 EV mRNA, CA19-9, CEA, DUPAN2 and Span-1, in PDAC and IPMN patients as compared to healthy controls. Table S7. Univariate analysis in PDAC patients in respect to survival (Exp. 2).
