# Supplementary material for: The Impact of Peroxiredoxin 3 on Molecular Testing, Diagnosis, and Prognosis in Human Pancreatic Ductal Adenocarcinoma
Source: Cancers (Basel). 2025 Jul 1;17(13):2212. doi: 10.3390/cancers17132212 (PMC12249400; doi:10.3390/cancers17132212)
Supplement: Supplementary file 1 [file cancers-17-02212-s001.zip › All Supplementary Tables and Figures.pdf]

Table S1. Clinicopathological characteristics of PDAC and IPMN patients (Exp. 1 and 2).

		PDAC (Exp. 1)			PDAC (Exp. 2)			IPMN (Exp. 2)		
		Median (Range)	*N*	%	Median (Range)	*N*	%	Median (Range)	*N*	%
**Patient demographics**										
Gender	Male		47	47.0		15	41.7		3	30.0
	Female		53	53.0		21	58.3		7	70.0
Age (y)	≤70	63.6 (40-70)	50	50.0	60.6 (44-70)	18	50.0	64.4 (55-70)	5	50.0
	>70	76.3 (71-87)	50	50.0	75.9 (71-85)	18	50.0	80.2 (71-87)	5	50.0
BMI	15-25	21.1 (15.8-24.8)	83	83.0	20.9 (17.5-24.9)	25	69.4	21.9 (17.9-24.6)	8	80.0
	>25	27.2 (25.1-30.5)	17	17.0	27.7 (25.2-31.0)	11	30.6	26.5 (25.1-28.1)	2	20.0
Diabetes	Negative		66	66.0		24	66.7		5	50.0
	Positive		34	34.0		12	33.3		5	50.0
Alcohol	Non-drinker		50	50.0		16	44.4		2	20.0
	Drinker		50	50.0		20	55.6		8	80.0
Smoking	Non-smoker		61	61.0		19	52.4		6	60.0
	Smoker		39	39.0		17	47.2		4	40.0
CA19-9	0-37 U/mL		24	24.0		8	22.2		10	100.0
	>37 U/mL		76	76.0		28	77.8		0	0.0
CEA	0-5.0 ng/mL		68	74.7		14	38.9		8	80.0
	>5.0 ng/mL		23	25.3		22	61.1		2	20.0
DUPAN-2	0-150 U/mL		43	56.6		5	29.4		4	100.0
	>150 U/mL		33	43.4		12	70.6		0	0.0
Span-1	0-30		36	37.9		9	34.6		5	100.0
	>30		59	62.1		17	65.4		0	0
**Tumor characteristics**										
Tumor size (cm)	≤2	1.6 (0.8-2.0)	23	23.0	0.9 (0.14-2.25)	5	13.9		-	-
	>2	3.3 (2.1-6.5)	77	77.0	46.6 (3.00-10.00)	31	86.1		-	-
Localization	Ph		56	56.0		25	69.4		-	-
	Phb		4	4.0		0	0.0		-	-
	Pb		21	21.0		4	11.1		-	-
	Pbt		11	11.0		0	0.0		-	-
	Pt		8	8.0		7	19.4		-	-
T factor	T1		13	13.0		2	5.6		-	-
	T2		32	32.0		2	5.6		-	-
	T3		44	44.0		17	47.2		-	-
	T4		11	11.0		15	41.7		-	-
N factor	N0		53	53.0		9	25.0		-	-
	N1		42	42.0		21	58.3		-	-
	N2		5	5.0		6	16.7		-	-
M factor	M0		99	99.0		22	61.1		-	-
	M1		1	1.0		14	38.9		-	-
Differentiation	Well		10	10.0		3	8.3		-	-
	Moderate		81	81.0		16	44.5		-	-
	Poor		9	9.0		17	47.2		-	-
Clinical stage	1		41	41.0		0	0.0		-	-
	2		47	47.0		5	13.9		-	-
	3		11	11.0		12	33.3		-	-
	4		1	1.0		19	52.8		-	-
Recurrence	Negative		30	30.0		2	10.5		-	-
	Positive		70	70.0		17	89.5		-	-
Lymphatic invasion (ly)	Negative		35	35.0		26	81.3		-	-
	Positive		65	65.0		6	18.8		-	-
Venous invasion (v)	Negative		62	62.0		6	80.6		-	-
	Positive		38	38.0		25	19.4		-	-
Invasive growth pattern (INF)	a		1	1.0		0	0.0		-	-
	b		86	86.0		17	53.1		-	-
	c		10	10.0		15	46.9		-	-

CA19-9, Cancer Antigen 19-9; CEA, Carcinoembryonic Antigen; DUPAN-2, Duke Pancreatic Monoclonal Antigen type 2; Span-1, S-pancreas-1 antigen

Table S2. Proteins up-regulated more than 5-fold in human PDAC detected by LC-MS/MS analysis

Symbol	Entrez Gene Name	Type(s)
ACSS3	acyl-CoA synthetase short chain family member 3	enzyme
AK1	adenylate kinase 1	kinase
AMPD2	adenosine monophosphate deaminase 2	enzyme
ANXA3	annexin A3	enzyme
ANXA10	annexin A10	other
ARF5	ADP ribosylation factor 5	enzyme
ARHGAP5	Rho GTPase activating protein 5	enzyme
ARPC1B	actin related protein 2/3 complex subunit 1B	other
ARMC4	armadillo repeat containing 4	other
BICD1	BICD cargo adaptor 1	other
BLVRB	biliverdin reductase B	enzyme
CAPZA1	capping actin protein of muscle Z-line subunit alpha 1	other
CAPZB	capping actin protein of muscle Z-line subunit beta	other
CASC4	cancer susceptibility 4	other
CBR3	carbonyl reductase 3	enzyme
CCT3	chaperonin containing TCP1 subunit 3	other
CLPX	caseinolytic mitochondrial matrix peptidase chaperone subunit	enzyme
CNN1	calponin 1	other
CNN3	calponin 3	other
COPG2	coatomer protein complex subunit gamma 2	transporter
CTSB	cathepsin B	peptidase
CTSZ	cathepsin Z	peptidase
DHDDS	dehydrodolichyl diphosphate synthase subunit	enzyme
DNAH2	dynein axonemal heavy chain 2	other
DNAJC27	DnaJ heat shock protein family (Hsp40) member C27	enzyme
DPYSL3	dihydropyrimidinase like 3	enzyme
DUSP13	dual specificity phosphatase 13	phosphatase
ECPAS	Ecm29 proteasome adaptor and scaffold	other
EIF4H	eukaryotic translation initiation factor 4H	TLR
ELAVL1	ELAV like RNA binding protein 1	other
ENAM	enamelin	other
EPB41L4B	erythrocyte membrane protein band 4.1 like 4B	transporter
FLNC	filamin C	other
FSCN1	fascin actin-bundling protein 1	other
FTH1	ferritin heavy chain 1	enzyme
GALNT17	polypeptide N-acetylgalactosaminyltransferase 17	enzyme
GAS8	growth arrest specific 8	other
GIMAP1	GTPase, IMAP family member 1	other
GNAI3	G protein subunit alpha i3	enzyme
GPCPD1	glycerophosphocholine phosphodiesterase 1	enzyme
GRB10	growth factor receptor bound protein 10	other
GSTP1	glutathione S-transferase pi 1	enzyme
INPP1	inositol polyphosphate-1-phosphatase	phosphatase
IPPK	inositol-pentakisphosphate 2-kinase	kinase
KANK4	KN motif and ankyrin repeat domains 4	other
KRT1	keratin 1	other
KRT13	keratin 13	other
KRT17	keratin 17	other
LASP1	LIM and SH3 protein 1	transporter
LMAN1	lectin, mannose binding 1	other
LMCD1	LIM and cysteine rich domains 1	TR
MVP	major vault protein	other
MAP4	microtubule associated protein 4	other
MAP1S	microtubule associated protein 1S	enzyme
MAP3K10	mitogen-activated protein kinase kinase kinase 10	kinase
MAPK15	mitogen-activated protein kinase 15	kinase

MCRIP2	MAPK regulated corepressor interacting protein 2	other
MCUB	mitochondrial calcium uniporter dominant negative beta subunit	other
ME1	malic enzyme 1	enzyme
MRPL9	mitochondrial ribosomal protein L9	TLR
MYL1	myosin light chain 1	other
MYO18B	myosin XVIIIB	other
NAIP	NLR family apoptosis inhibitory protein	other
NAP1L4	nucleosome assembly protein 1 like 4	other
NF1	neurofibromin 1	other
NPEPPS	aminopeptidase puromycin sensitive	peptidase
OXCT2	3-oxoacid CoA-transferase 2	enzyme
PATL1	PAT1 homolog 1, processing body mRNA decay factor	TLR
PDE6C	phosphodiesterase 6C	enzyme
PDLIM7	PDZ and LIM domain 7	other
PEX5	peroxisomal biogenesis factor 5	other
PIWIL4	piwi like RNA-mediated gene silencing 4	other
PLS3	plastin 3	other
PNML1	PNMA family member L1	other
PRX3	peroxiredoxin 3	enzyme
PSMB9	proteasome subunit beta 9	peptidase
PSME1	proteasome activator subunit 1	other
PSRC1	proline and serine rich coiled-coil 1	other
PSTPIP1	proline-serine-threonine phosphatase interacting protein 1	other
PXK	PX domain containing serine/threonine kinase like	kinase
RHOT1	ras homolog family member T1	enzyme
RNASET2	ribonuclease T2	enzyme
ROCK1	Rho associated coiled-coil containing protein kinase 1	kinase
S100A8	S100 calcium binding protein A8	other
S100A11	S100 calcium binding protein A11	other
S100P	S100 calcium binding protein P	other
SHB	SH2 domain containing adaptor protein B	other
SHMT2	serine hydroxymethyltransferase 2	enzyme
SLC25A3	solute carrier family 25 member 3	transporter
SLC26A6	solute carrier family 26 member 6	transporter
SYNJ2	synaptojanin 2	phosphatase
TBC1D4	TBC1 domain family member 4	other
TNRC6B	trinucleotide repeat containing adaptor 6B	other
TRAP1	TNF receptor associated protein 1	enzyme
TRIP11	thyroid hormone receptor interactor 11	TR
TRIP12	thyroid hormone receptor interactor 12	enzyme
TSPOAP1	TSPO associated protein 1	other
TTC28	tetratricopeptide repeat domain 28	other
TUBA3E	tubulin alpha 3e	other
TXN	thioredoxin	enzyme
UBA1	ubiquitin like modifier activating enzyme 1	enzyme
UGGT1	UDP-glucose glycoprotein glucosyltransferase 1	enzyme
UGP2	UDP-glucose pyrophosphorylase 2	enzyme
WDR45	WD repeat domain 45	other
XYLT1	xylosyltransferase 1	enzyme
ZFYVE28	zinc finger FYVE-type containing 28	other

Table S3. Biomarker Filter Analysis by IPA

Symbol	Entrez Gene Name	Location	Type	Function
PRDX3	peroxiredoxin 3	Cytoplasm	enzyme	mitochondrial antioxidant
TXN	thioredoxin	Cytoplasm	enzyme	antioxidant and redox signaling
ARMC4	armadillo repeat containing 4	Extracellular Space	other	cell growth and survival
PNMAL1	PNMA family member L1	Cytoplasm	other	neuron-specific protein
DPYSL3	dihydropyrimidinase like 3	Cytoplasm	enzyme	neurogenesis
MVP	major vault protein	Cytoplasm	other	immune response

© 2000-2024 QIAGEN. All rights reserved.

Table S4. Canonical Pathway analysis by IPA

Ingenuity Canonical Pathways	-log (p-value)	z-score
Fibrogenesis	10.6	4.1
GP6 Signaling Pathway	9.4	3.7
Actin Cytoskeleton Signaling	4.76	2.7
Signaling by Rho Family GTPases	4.27	2.8
Regulation of Actin-based Motility by Rho	3.13	2.2
RHOA Signaling	2.67	2.0
Production of Nitric Oxide and Reactive Oxygen Species	5.79	2.8
NRF2-mediated Oxidative Stress Response	0.81	2.0
Integrin Signaling	2.57	2.3
HIF1α Signaling	0.623	2.0
Phagosome Formation	2.39	4.0
Fcγ Receptor-mediated Phagocytosis in Macrophages and Monocytes	1.58	2.0
IL-8 Signaling	1.39	2.5
IL-12 Signaling and Production in Macrophages	2.23	2.0
IL-13 Signaling Pathway	1.3	2.0
CXCR4 Signaling	0.842	2.0
LXR/RXR Activation	2.54	2.3
CXCR4 Signaling	0.842	2.0
Glycolysis I	2.29	2.0
Gluconeogenesis I	2.4	2.0

© 2000-2024 QIAGEN. All rights reserved.

Table S5. Univariate and multivariate analyses in PDAC patients in respect to survival (**Exp.1**)

	UA			MA		
Clinicopathological features	Hazard ratio	95% CI	*P*	Hazard ratio	95% CI	*P*
Age						
≤70 vs >70	1.212	0.729-2.016	0.459			NA
Smoking						
Positive vs Negative	1.481	0.889-2.467	0.131			NA
Drinking						
Positive vs Negative	0.857	0.515-1.428	0.555			NA
Diabetes						
Positive vs Negative	1.058	0.611-1.833	0.840			NA
Tumor size						
>4 cm vs ≤4 cm	1.907	1.009-3.606	**0.047**			NA
Lymphatic invasion (ly)						
Positive vs negative	2.836	1.272-6.322	**0.011**	0.291	0.087-0.978	**0.046**
Venous invasion (v)						
Positive vs negative	2.511	0.861-7.324	0.082			NA
Infiltrative growth pattern						
INFc vs INFab	2.141	1.658-3.609	**0.048**			NA
pA						
Positive vs Negative	2.432	1.102-5.365	**0.028**			NA
pPL						
Positive vs Negative	1.888	0.984-3.623	0.056			NA
Differentiation						
Poor vs Well & Moderate	2.432	1.094-5.402	**0.029**			NA
T category						
T3,4 vs T1,2	2.035	1.153-3.590	**0.014**			NA
pN						
N1-2 vs N0	1.648	0.989-2.746	0.050			NA
Stage						
2-4 vs 1	1.904	1.109-3.272	**0.020**			NA
CA19-9						
>35 vs ≤35	2.192	1.132-4.248	**0.020**	0.207	0.053-0.804	**0.023**
CA19-9+PRX3						
High vs Low	1.877	1.121-3.143	**0.017**			NA
CEA						
>5 vs ≤5	1.353	0.735-2.491	0.352			NA
CEA+PRX3						
High vs Low	1.401	0.684-2.870	0.357			NA
DUPAN-2						
>150 vs ≤150	1.381	0.763-2.502	0.287			NA
DUPAN-2+PRX3						
High vs Low	1.950	0.995-3.820	**0.049**			NA
Span-1						
>30 vs ≤30	1.729	0.993-3.010	0.050			NA
Span-1+PRX3						
High vs Low	2.287	1.331-3.929	**0.003**	0.176	0.045-0.689	**0.013**

Table S6. Sensitivity, Specificity and AUC values for PRX3 protein, PRX3 EV mRNA, CA19-9, CEA, DUPAN2 and Span-1, in PDAC and IPMN patients as compared to healthy controls

	Outcome	AUC	Cut-off point	Sensitivity (%)	Specificity (%)
PRX3 protein (ng/ml)	PDAC	0.950	140.0	75.0	80.0
	IPMN	0.820	140.0	50.0	80.0
PRX3 EV mRNA (PRDX3/18S)	PDAC	0.822	0.01	63.9	80.0
	IPMN*	ND	ND	ND	ND
CA19-9 (U/ml)	PDAC	0.980	44.5	90.0	80.0
	IPMN*	ND	ND	ND	ND
CEA (U/ml)	PDAC	0.856	5.1	61.1	100.0
	IPMN*	ND	ND	ND	ND
DUPAN-2 (U/ml)	PDAC	0.935	155.0	64.7	100.0
	IPMN*	ND	ND	ND	ND
Span-1 (U/ml)	PDAC	0.996	29.5	69.2	100.0
	IPMN*	ND	ND	ND	ND

*, no significant increase was detected as compared to healthy control subjects

Table S7. Univariate analysis in PDAC patients in respect to survival (**Exp.2**).

	UA			MA		
Clinicopathological features	Hazard ratio	95% CI	*P*	Hazard ratio	95% CI	*P*
PRX3 EV mRN. >0.01PRX3/18S High vs Low	5.123	1.484-17.684	**0.010**	0.069	0.012-0.381	**0.002**
PRX3 protein >140ng/mL High vs Low	1.049	0.398-2.765	0.923			
pN N1,2 vs N0	11.143	1.473-84.270	**0.020**	**0.022**	0.001-0.420	**0.011**
Differentiation Poor vs Well & Moderate	4.234	1.596-11.236	**0.004**	0.215	0.056-0.821	**0.025**
T category T4 vs T1,2,3	4.826	1.882-12.375	**0.001**			NA
M M1 vs M0	7.283	2.759-19.224	**0.000**			NA
Stage 4 vs I,2,3	3.589	1.377-9.355	**0.009**			NA
Invasive growth mode INFc vs INFab	4.555	1.620-12.811	**0.004**			NA
Tumor size >4 cm vs ≤4 cm	5.561	2.163-14.296	**0.000**			NA

NA: not available; UA: Univariate analysis, Cox proportional-hazards regression;
MA: Multivariate analysis, Cox proportional-hazards regression; variables were adopted in multivariate analysis for their prognostic significance by univariate analysis.

Supplementary Figures

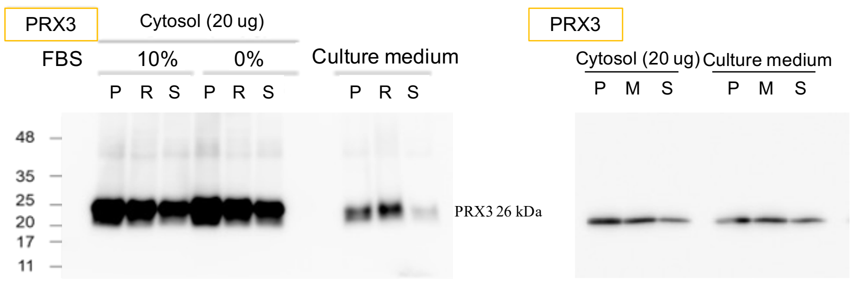

Figure S1. Secretion of PRX3 protein to culture medium of PANC-1 (P), RWP-1 (R), SW1990 (S), and MIAPaCa-2 (M) PA cells

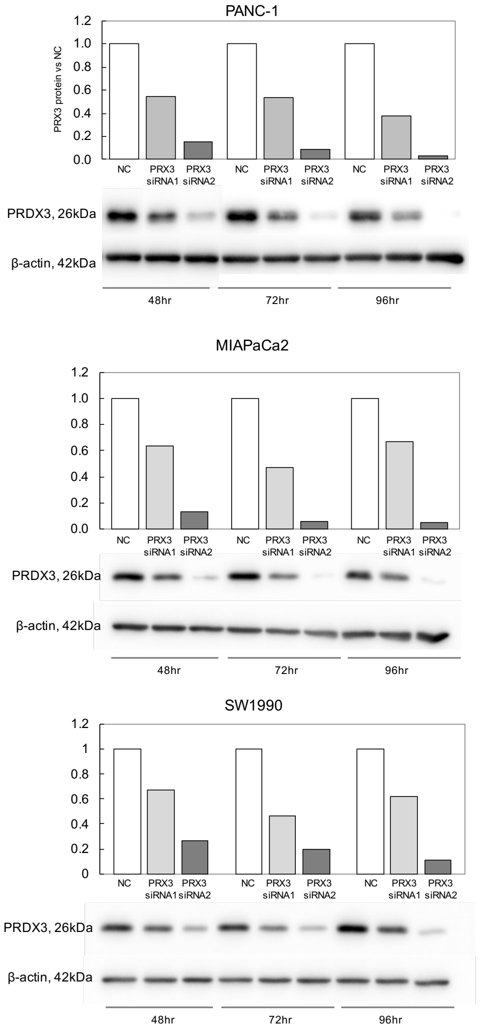

Figure S2. PRX3 siRNA knockdown in PANC-1, MIA-PaCa-2, and SW1990 cells Western blots confirming the PRX3 silencing. Best results were obtained for PRX3 siRNA2.

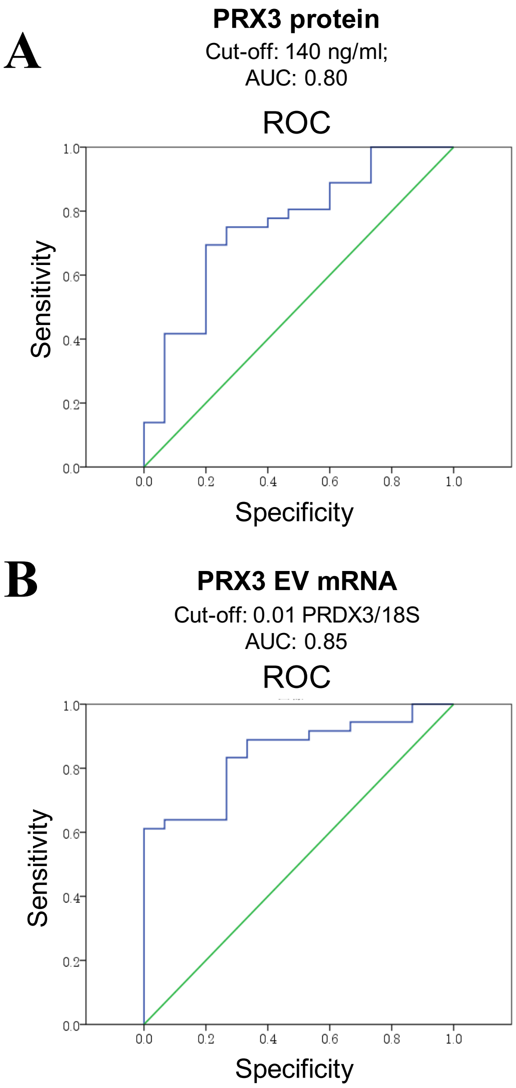

Figure S3. ROC curves for the prediction of PDAC differentiating from combined IPMN and control subjects based on the serum levels of PRX3 protein (A) and its EV mRNA (B) measured by ELISA and quantitative RT-PCR, respectively.
