# Supplementary material for: The Impact of Peroxiredoxin 3 on Molecular Testing, Diagnosis, and Prognosis in Human Pancreatic Ductal Adenocarcinoma
Source: Cancers (Basel). 2025 Jul 1;17(13):2212. doi: 10.3390/cancers17132212 (PMC12249400; doi:10.3390/cancers17132212)
Supplement: Supplementary file 1 [file cancers-17-02212-s001.zip › Figure S2.pdf]

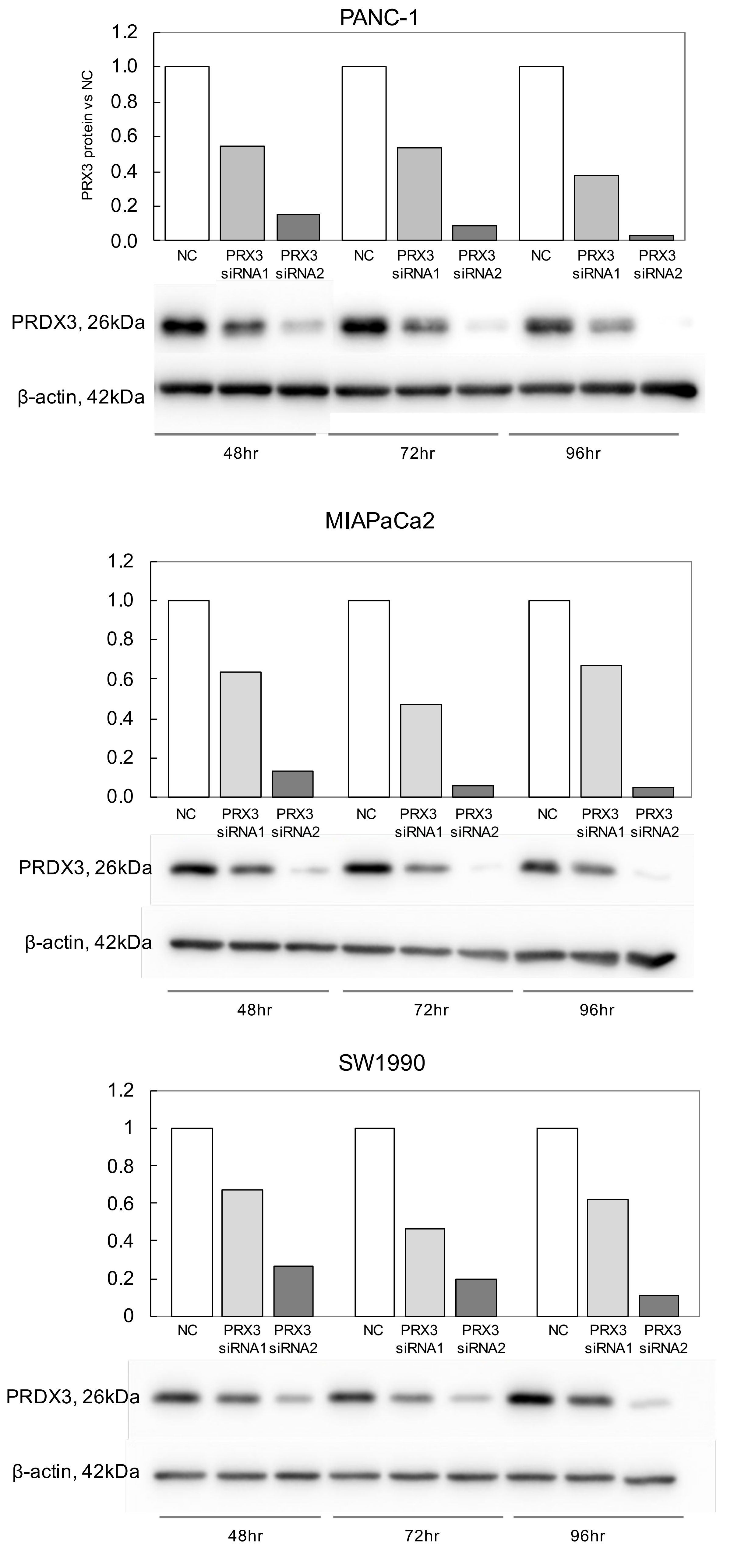

Figure S2. PRX3 siRNA knockdown in PANC-1, MIA-PaCa-2, and SW1990 cells Western blots confirming the PRX3 silencing. Best results were obtained for PRX3 siRNA2.
