# Supplementary material for: The Impact of Peroxiredoxin 3 on Molecular Testing, Diagnosis, and Prognosis in Human Pancreatic Ductal Adenocarcinoma
Source: Cancers (Basel). 2025 Jul 1;17(13):2212. doi: 10.3390/cancers17132212 (PMC12249400; doi:10.3390/cancers17132212)
Supplement: Supplementary file 1 [file cancers-17-02212-s001.zip › Figure S3.pdf]

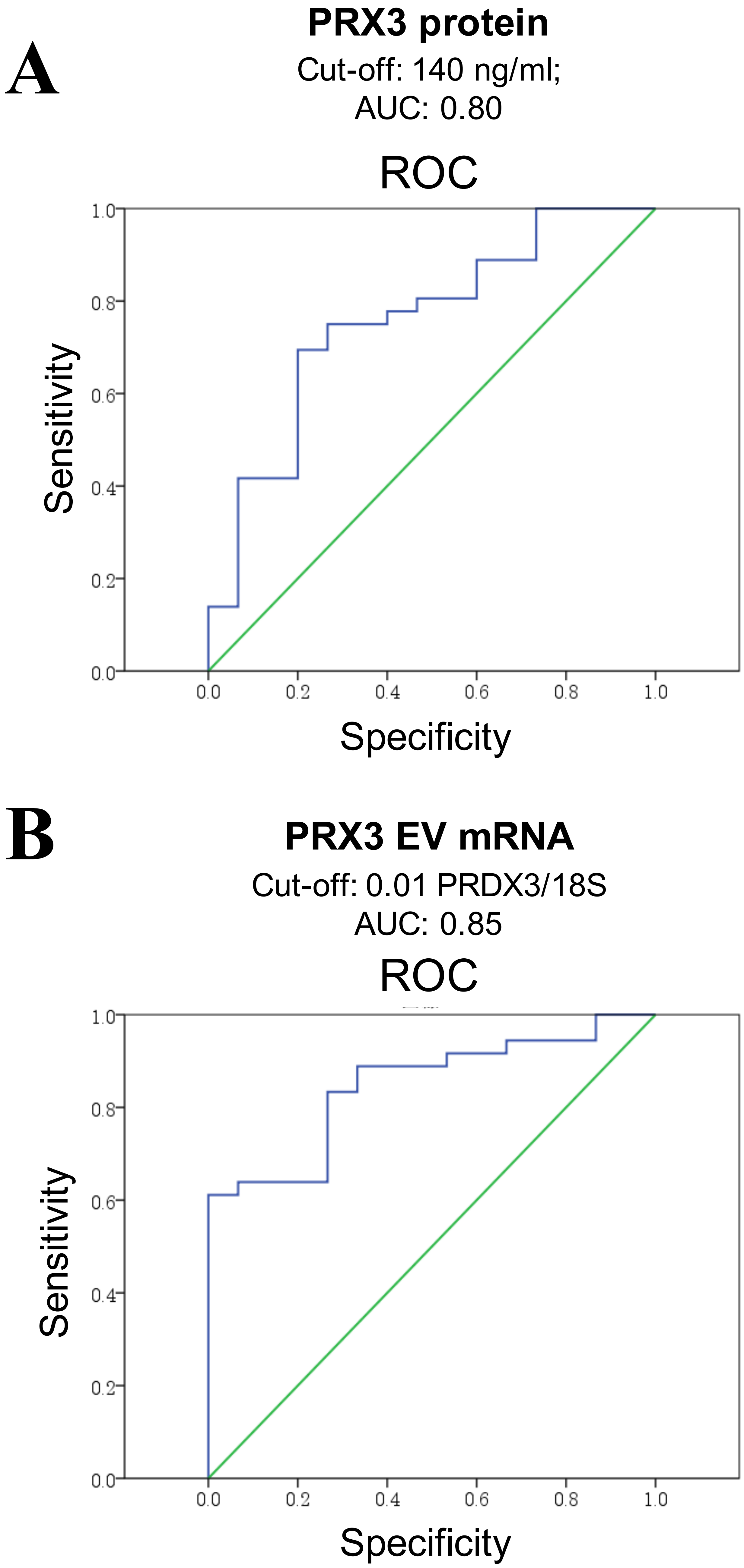

Figure S3. ROC curves for the prediction of PDAC differentiating from combined IPMN and control subjects based on the serum levels of PRX3 protein (A) and its EV mRNA (B) measured by ELISA and quantitative RT-PCR, respectively.
