# Supplementary material for: The Impact of Peroxiredoxin 3 on Molecular Testing, Diagnosis, and Prognosis in Human Pancreatic Ductal Adenocarcinoma
Source: Cancers (Basel). 2025 Jul 1;17(13):2212. doi: 10.3390/cancers17132212 (PMC12249400; doi:10.3390/cancers17132212)
Supplement: Supplementary file 1 [file cancers-17-02212-s001.zip › Table S1 .pdf]

Table S1. Clinicopathological characteristics of PDAC and IPMN patients (Exp. 1 and 2).

		PDAC (Exp. 1)			PDAC (Exp. 2)			IPMN (Exp. 2)		
		Median (Range)	*N*	%	Median (Range)	*N*	%	Median (Range)	*N*	%
**Patient demographics**										
Gender	Male		47	47.0		15	41.7		3	30.0
	Female		53	53.0		21	58.3		7	70.0
Age (y)	≤70	63.6 (40-70)	50	50.0	60.6 (44-70)	18	50.0	64.4 (55-70)	5	50.0
	>70	76.3 (71-87)	50	50.0	75.9 (71-85)	18	50.0	80.2 (71-87)	5	50.0
BMI	15-25	21.1 (15.8-24.8)	83	83.0	20.9 (17.5-24.9)	25	69.4	21.9 (17.9-24.6)	8	80.0
	>25	27.2 (25.1-30.5)	17	17.0	27.7 (25.2-31.0)	11	30.6	26.5 (25.1-28.1)	2	20.0
Diabetes	Negative		66	66.0		24	66.7		5	50.0
	Positive		34	34.0		12	33.3		5	50.0
Alcohol	Non-drinker		50	50.0		16	44.4		2	20.0
	Drinker		50	50.0		20	55.6		8	80.0
Smoking	Non-smoker		61	61.0		19	52.4		6	60.0
	Smoker		39	39.0		17	47.2		4	40.0
CA19-9	0-37 U/mL		24	24.0		8	22.2		10	100.0
	>37 U/mL		76	76.0		28	77.8		0	0.0
CEA	0-5.0 ng/mL		68	74.7		14	38.9		8	80.0
	>5.0 ng/mL		23	25.3		22	61.1		2	20.0
DUPAN-2	0-150 U/mL		43	56.6		5	29.4		4	100.0
	>150 U/mL		33	43.4		12	70.6		0	0.0
Span-1	0-30		36	37.9		9	34.6		5	100.0
	>30		59	62.1		17	65.4		0	0
**Tumor characteristics**										
Tumor size (cm)	≤2	1.6 (0.8-2.0)	23	23.0	0.9 (0.14-2.25)	5	13.9		-	-
	>2	3.3 (2.1-6.5)	77	77.0	46.6 (3.00-10.00)	31	86.1		-	-
Localization	Ph		56	56.0		25	69.4		-	-
	Phb		4	4.0		0	0.0		-	-
	Pb		21	21.0		4	11.1		-	-
	Pbt		11	11.0		0	0.0		-	-
	Pt		8	8.0		7	19.4		-	-
T factor	T1		13	13.0		2	5.6		-	-
	T2		32	32.0		2	5.6		-	-
	T3		44	44.0		17	47.2		-	-
	T4		11	11.0		15	41.7		-	-
N factor	N0		53	53.0		9	25.0		-	-
	N1		42	42.0		21	58.3		-	-
	N2		5	5.0		6	16.7		-	-
M factor	M0		99	99.0		22	61.1		-	-
	M1		1	1.0		14	38.9		-	-
Differentiation	Well		10	10.0		3	8.3		-	-
	Moderate		81	81.0		16	44.5		-	-
	Poor		9	9.0		17	47.2		-	-
Clinical stage	1		41	41.0		0	0.0		-	-
	2		47	47.0		5	13.9		-	-
	3		11	11.0		12	33.3		-	-
	4		1	1.0		19	52.8		-	-
Recurrence	Negative		30	30.0		2	10.5		-	-
	Positive		70	70.0		17	89.5		-	-
Lymphatic invasion	Negative		35	35.0		26	81.3		-	-
(ly)	Positive		65	65.0		6	18.8		-	-
Venous invasion	Negative		62	62.0		6	80.6		-	-
(v)	Positive		38	38.0		25	19.4		-	-
Invasive growth	a		1	1.0		0	0.0		-	-
pattern (INF)	b		86	86.0		17	53.1		-	-
	c		10	10.0		15	46.9		-	-

CA19-9, Cancer Antigen 19-9; CEA, Carcinoembryonic Antigen; DUPAN-2, Duke Pancreatic Monoclonal Antigen type 2; Span-1, S-pancreas-1 antigen
