# Supplementary material for: The Impact of Peroxiredoxin 3 on Molecular Testing, Diagnosis, and Prognosis in Human Pancreatic Ductal Adenocarcinoma
Source: Cancers (Basel). 2025 Jul 1;17(13):2212. doi: 10.3390/cancers17132212 (PMC12249400; doi:10.3390/cancers17132212)
Supplement: Supplementary file 1 [file cancers-17-02212-s001.zip › Table S2.pdf]

Table S2. Proteins up-regulated more than 5-fold in human PDAC detected by LC-MS/MS analysis

Symbol	Entrez Gene Name	Type(s)
ACSS3	acyl-CoA synthetase short chain family member 3	enzyme
AK1	adenylate kinase 1	kinase
AMPD2	adenosine monophosphate deaminase 2	enzyme
ANXA3	annexin A3	enzyme
ANXA10	annexin A10	other
ARF5	ADP ribosylation factor 5	enzyme
ARHGAP5	Rho GTPase activating protein 5	enzyme
ARPC1B	actin related protein 2/3 complex subunit 1B	other
ARMC4	armadillo repeat containing 4	other
BICD1	BICD cargo adaptor 1	other
BLVRB	biliverdin reductase B	enzyme
CAPZA1	capping actin protein of muscle Z-line subunit alpha 1	other
CAPZB	capping actin protein of muscle Z-line subunit beta	other
CASC4	cancer susceptibility 4	other
CBR3	carbonyl reductase 3	enzyme
CCT3	chaperonin containing TCP1 subunit 3	other
CLPX	caseinolytic mitochondrial matrix peptidase chaperone subunit	enzyme
CNN1	calponin 1	other
CNN3	calponin 3	other
COPG2	coatomer protein complex subunit gamma 2	transporter
CTSB	cathepsin B	peptidase
CTSZ	cathepsin Z	peptidase
DHDDS	dehydrodolichyl diphosphate synthase subunit	enzyme
DNAH2	dynein axonemal heavy chain 2	other
DNAJC27	DnaJ heat shock protein family (Hsp40) member C27	enzyme
DPYSL3	dihydropyrimidinase like 3	enzyme
DUSP13	dual specificity phosphatase 13	phosphatase
ECPAS	Ecm29 proteasome adaptor and scaffold	other
EIF4H	eukaryotic translation initiation factor 4H	TLR
ELAVL1	ELAV like RNA binding protein 1	other
ENAM	enamelin	other
EPB41L4B	erythrocyte membrane protein band 4.1 like 4B	transporter
FLNC	filamin C	other
FSCN1	fascin actin-bundling protein 1	other
FTH1	ferritin heavy chain 1	enzyme
GALNT17	polypeptide N-acetylgalactosaminyltransferase 17	enzyme
GAS8	growth arrest specific 8	other
GIMAP1	GTPase, IMAP family member 1	other
GNAI3	G protein subunit alpha i3	enzyme
GPCPD1	glycerophosphocholine phosphodiesterase 1	enzyme
GRB10	growth factor receptor bound protein 10	other
GSTP1	glutathione S-transferase pi 1	enzyme
INPP1	inositol polyphosphate-1-phosphatase	phosphatase
IPPK	inositol-pentakisphosphate 2-kinase	kinase
KANK4	KN motif and ankyrin repeat domains 4	other
KRT1	keratin 1	other
KRT13	keratin 13	other
KRT17	keratin 17	other
LASP1	LIM and SH3 protein 1	transporter
LMAN1	lectin, mannose binding 1	other
LMCD1	LIM and cysteine rich domains 1	TR
MVP	major vault protein	other
MAP4	microtubule associated protein 4	other
MAP1S	microtubule associated protein 1S	enzyme
MAP3K10	mitogen-activated protein kinase kinase kinase 10	kinase
MAPK15	mitogen-activated protein kinase 15	kinase
MCRIP2	MAPK regulated corepressor interacting protein 2	other
MCUB	mitochondrial calcium uniporter dominant negative beta subunit	other
ME1	malic enzyme 1	enzyme
MRPL9	mitochondrial ribosomal protein L9	TLR

MYL1	myosin light chain 1	other
MYO18B	myosin XVIIIB	other
NAIP	NLR family apoptosis inhibitory protein	other
NAP1L4	nucleosome assembly protein 1 like 4	other
NF1	neurofibromin 1	other
NPEPPS	aminopeptidase puromycin sensitive	peptidase
OXCT2	3-oxoacid CoA-transferase 2	enzyme
PATL1	PAT1 homolog 1, processing body mRNA decay factor	TLR
PDE6C	phosphodiesterase 6C	enzyme
PDLIM7	PDZ and LIM domain 7	other
PEX5	peroxisomal biogenesis factor 5	other
PIWIL4	piwi like RNA-mediated gene silencing 4	other
PLS3	plastin 3	other
PNML1	PNMA family member L1	other
PRX3	peroxiredoxin 3	enzyme
PSMB9	proteasome subunit beta 9	peptidase
PSME1	proteasome activator subunit 1	other
PSRC1	proline and serine rich coiled-coil 1	other
PSTPIP1	proline-serine-threonine phosphatase interacting protein 1	other
PXK	PX domain containing serine/threonine kinase like	kinase
RHOT1	ras homolog family member T1	enzyme
RNASET2	ribonuclease T2	enzyme
ROCK1	Rho associated coiled-coil containing protein kinase 1	kinase
S100A8	S100 calcium binding protein A8	other
S100A11	S100 calcium binding protein A11	other
S100P	S100 calcium binding protein P	other
SHB	SH2 domain containing adaptor protein B	other
SHMT2	serine hydroxymethyltransferase 2	enzyme
SLC25A3	solute carrier family 25 member 3	transporter
SLC26A6	solute carrier family 26 member 6	transporter
SYNJ2	synaptojanin 2	phosphatase
TBC1D4	TBC1 domain family member 4	other
TNRC6B	trinucleotide repeat containing adaptor 6B	other
TRAP1	TNF receptor associated protein 1	enzyme
TRIP11	thyroid hormone receptor interactor 11	TR
TRIP12	thyroid hormone receptor interactor 12	enzyme
TSPOAP1	TSPO associated protein 1	other
TTC28	tetratricopeptide repeat domain 28	other
TUBA3E	tubulin alpha 3e	other
TXN	thioredoxin	enzyme
UBA1	ubiquitin like modifier activating enzyme 1	enzyme
UGGT1	UDP-glucose glycoprotein glucosyltransferase 1	enzyme
UGP2	UDP-glucose pyrophosphorylase 2	enzyme
WDR45	WD repeat domain 45	other
XYLT1	xylosyltransferase 1	enzyme
ZFYVE28	zinc finger FYVE-type containing 28	other
