# Supplementary material for: The Impact of Peroxiredoxin 3 on Molecular Testing, Diagnosis, and Prognosis in Human Pancreatic Ductal Adenocarcinoma
Source: Cancers (Basel). 2025 Jul 1;17(13):2212. doi: 10.3390/cancers17132212 (PMC12249400; doi:10.3390/cancers17132212)
Supplement: Supplementary file 1 [file cancers-17-02212-s001.zip › Table S3.pdf]

Table S3. Biomarker Filter Analysis by IPA

Symbol	Entrez Gene Name	Location	Type	Function
PRDX3	peroxiredoxin 3	Cytoplasm	enzyme	mitochondrial antioxidant
TXN	thioredoxin	Cytoplasm	enzyme	antioxidant and redox signaling
ARMC4	armadillo repeat containing 4	Extracellular Space	other	cell growth and survival
PNMAL1	PNMA family member L1	Cytoplasm	other	neuron-specific protein
DPYSL3	dihydropyrimidinase like 3	Cytoplasm	enzyme	neurogenesis
MVP	major vault protein	Cytoplasm	other	immune response

© 2000-2024 QIAGEN. All rights reserved.
