# Supplementary material for: The Impact of Peroxiredoxin 3 on Molecular Testing, Diagnosis, and Prognosis in Human Pancreatic Ductal Adenocarcinoma
Source: Cancers (Basel). 2025 Jul 1;17(13):2212. doi: 10.3390/cancers17132212 (PMC12249400; doi:10.3390/cancers17132212)
Supplement: Supplementary file 1 [file cancers-17-02212-s001.zip › Table S4 .pdf]

Table S4. Canonical Pathway analysis by IPA

Ingenuity Canonical Pathways	-log (p-value)	z-score
Fibrogenesis	10.6	4.1
GP6 Signaling Pathway	9.4	3.7
Actin Cytoskeleton Signaling	4.76	2.7
Signaling by Rho Family GTPases	4.27	2.8
Regulation of Actin-based Motility by Rho	3.13	2.2
RHOA Signaling	2.67	2.0
Production of Nitric Oxide and Reactive Oxygen Species	5.79	2.8
NRF2-mediated Oxidative Stress Response	0.81	2.0
Integrin Signaling	2.57	2.3
HIF1α Signaling	0.623	2.0
Phagosome Formation	2.39	4.0
Fcγ Receptor-mediated Phagocytosis in Macrophages and Monocytes	1.58	2.0
IL-8 Signaling	1.39	2.5
IL-12 Signaling and Production in Macrophages	2.23	2.0
IL-13 Signaling Pathway	1.3	2.0
CXCR4 Signaling	0.842	2.0
LXR/RXR Activation	2.54	2.3
CXCR4 Signaling	0.842	2.0
Glycolysis I	2.29	2.0
Gluconeogenesis I	2.4	2.0

© 2000-2024 QIAGEN. All rights reserved.
