# Supplementary material for: The Impact of Peroxiredoxin 3 on Molecular Testing, Diagnosis, and Prognosis in Human Pancreatic Ductal Adenocarcinoma
Source: Cancers (Basel). 2025 Jul 1;17(13):2212. doi: 10.3390/cancers17132212 (PMC12249400; doi:10.3390/cancers17132212)
Supplement: Supplementary file 1 [file cancers-17-02212-s001.zip › Table S5.pdf]

Table S5. Univariate and multivariate analyses in PDAC patients in respect to survival (**Exp.1**)

Clinicopathological features	UA			MA		
	Hazard ratio	95% CI	*P*	Hazard ratio	95% CI	*P*
Age						
≤70 vs >70	1.212	0.729-2.016	0.459			NA
Smoking						
Positive vs Negative	1.481	0.889-2.467	0.131			NA
Drinking						
Positive vs Negative	0.857	0.515-1.428	0.555			NA
Diabetes						
Positive vs Negative	1.058	0.611-1.833	0.840			NA
Tumor size						
>4 cm vs ≤4 cm	1.907	1.009-3.606	**0.047**			NA
Lymphatic invasion (ly)						
Positive vs negative	2.836	1.272-6.322	**0.011**	0.291	0.087-0.978	**0.046**
Venous invasion (v)						
Positive vs negative	2.511	0.861-7.324	0.082			NA
Infiltrative growth pattern						
INFc vs INFab	2.141	1.658-3.609	**0.048**			NA
pA						
Positive vs Negative	2.432	1.102-5.365	**0.028**			NA
pPL						
Positive vs Negative	1.888	0.984-3.623	0.056			NA
Differentiation						
Poor vs Well & Moderate	2.432	1.094-5.402	**0.029**			NA
T category						
T3,4 vs T1,2	2.035	1.153-3.590	**0.014**			NA
pN						
N1-2 vs N0	1.648	0.989-2.746	0.050			NA
Stage						
2-4 vs 1	1.904	1.109-3.272	**0.020**			NA
CA19-9						
>35 vs ≤35	2.192	1.132-4.248	**0.020**	0.207	0.053-0.804	**0.023**
CA19-9+PRX3						
High vs Low	1.877	1.121-3.143	**0.017**			NA
CEA						
>5 vs ≤5	1.353	0.735-2.491	0.352			NA
CEA+PRX3						
High vs Low	1.401	0.684-2.870	0.357			NA
DUPAN-2						
>150 vs ≤150	1.381	0.763-2.502	0.287			NA
DUPAN-2+PRX3						
High vs Low	1.950	0.995-3.820	**0.049**			NA
Span-1						
>30 vs ≤30	1.729	0.993-3.010	0.050			NA
Span-1+PRX3						
High vs Low	2.287	1.331-3.929	**0.003**	0.176	0.045-0.689	**0.013**

Abbreviations: CA19-9; Carbohydrate antigen 19-9; CEA: carcinoembryonic antigen; DUPAN-2: Duke pancreatic mono-clonal antigen type 2; Span-1: S-Pancreas-1 Antigen; NA: not available;
UA: Univariate analysis, Cox proportional-hazards regression;
MA: Multivariate analysis, Cox proportional-hazards regression; variables were adopted in multivariate analysis for their prognostic significance by univariate analysis.
