# Supplementary material for: The Impact of Peroxiredoxin 3 on Molecular Testing, Diagnosis, and Prognosis in Human Pancreatic Ductal Adenocarcinoma
Source: Cancers (Basel). 2025 Jul 1;17(13):2212. doi: 10.3390/cancers17132212 (PMC12249400; doi:10.3390/cancers17132212)
Supplement: Supplementary file 1 [file cancers-17-02212-s001.zip › Table S6 biomarker performance.pdf]

Table S6. Sensitivity, Specificity and AUC values for PRX3 protein, PRX3 EV mRNA, CA19-9, CEA, DUPAN2 and Span-1, in PDAC and IPMN patients as compared to healthy controls

	Outcome	AUC	Cut-off point	Sensitivity (%)	Specificity (%)
PRX3 protein (ng/ml)	PDAC	0.950	140.0	75.0	80.0
	IPMN	0.820	140.0	50.0	80.0
PRX3 EV mRNA (PRDX3/18S)	PDAC	0.822	0.01	63.9	100.0
	IPMN*	ND	ND	ND	ND
CA19-9 (U/ml)	PDAC	0.980	44.5	90.0	80.0
	IPMN*	ND	ND	ND	ND
CEA (U/ml)	PDAC	0.856	5.1	61.1	100.0
	IPMN*	ND	ND	ND	ND
DUPAN-2 (U/ml)	PDAC	0.935	155.0	64.7	100.0
	IPMN*	ND	ND	ND	ND
Span-1 (U/ml)	PDAC	0.996	29.5	69.2	100.0
	IPMN*	ND	ND	ND	ND

*, no significant increase was detected as compared to healthy control subjects
