# Supplementary material for: The Impact of Peroxiredoxin 3 on Molecular Testing, Diagnosis, and Prognosis in Human Pancreatic Ductal Adenocarcinoma
Source: Cancers (Basel). 2025 Jul 1;17(13):2212. doi: 10.3390/cancers17132212 (PMC12249400; doi:10.3390/cancers17132212)
Supplement: Supplementary file 1 [file cancers-17-02212-s001.zip › Table S7 Blood data univariate analysis without mixed markers.pdf]

Table S7. Univariate analysis in PDAC patients in respect to survival (Exp.2).

	UA			MA		
Clinicopathological features	Hazard ratio	95% CI	*P*	Hazard ratio	95% CI	*P*
PRX3 EV mRNA >0.01PRX3/18S High vs Low	5.123	1.484-17.684	**0.010**	0.069	0.012-0.381	**0.002**
PRX3 protein >140ng/mL						
High vs Low	1.049	0.398-2.765	0.923			
pN						
N1,2 vs N0	11.143	1.473-84.270	**0.020**	**0.022**	0.001-0.420	**0.011**
Differentiation						
Poor vs Well & Moderate	4.234	1.596-11.236	**0.004**	0.215	0.056-0.821	**0.025**
T category						
T4 vs T1,2,3	4.826	1.882-12.375	**0.001**			NA
M						
M1 vs M0	7.283	2.759-19.224	**0.000**			NA
Stage						
4 vs I,2,3	3.589	1.377-9.355	**0.009**			NA
Invasive growth mode						
INFc vs INFab	4.555	1.620-12.811	**0.004**			NA
Tumor size						
>4 cm vs ≤4 cm	5.561	2.163-14.296	**0.000**			NA

NA: not available; UA: Univariate analysis, Cox proportional-hazards regression;
MA: Multivariate analysis, Cox proportional-hazards regression; variables were adopted in multivariate analysis for their prognostic significance by univariate analysis.

Abbreviations: UA: Univariate analysis; CA19-9; Carbohydrate antigen 19-9;
CEA: carcinoembryonic antigen; DUPAN-2: Duke pancreatic mono-clonal antigen type 2;
Span-1: S-Pancreas-1 Antigen
