# Supplementary figures and images for: The Impact of Peroxiredoxin 3 on Molecular Testing, Diagnosis, and Prognosis in Human Pancreatic Ductal Adenocarcinoma
Source: Cancers (Basel). 2025 Jul 1;17(13):2212. doi: 10.3390/cancers17132212 (PMC12249400; doi:10.3390/cancers17132212)

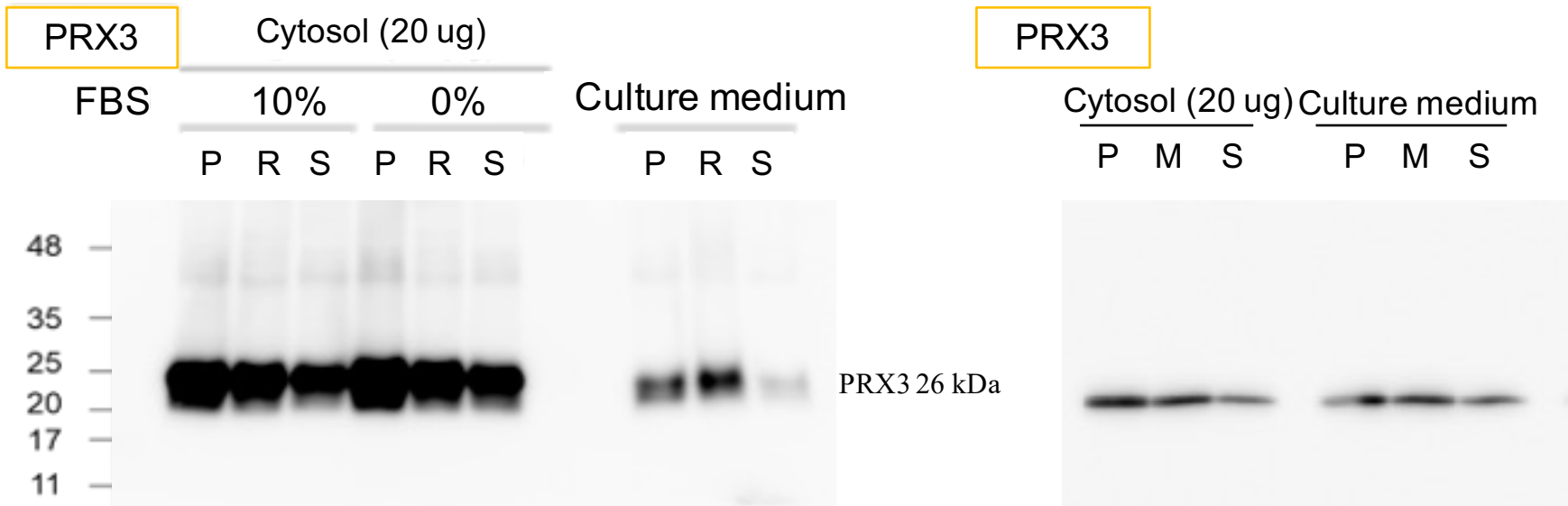

Figure S1. Secretion of PRX3 protein to culture medium of PANC-1 (P), RWP-1 (R), SW1990 (S), and MIAPaCa-2 (M) PA cells

Supplement: Supplementary file 1 [file cancers-17-02212-s001.zip › Figure S1.pdf]
